# Self-Oriented Gradient Ionic Skins for Dual-Function Electromagnetic Shielding and Self-Powered Sensing

**DOI:** 10.1007/s40820-026-02255-z

**Published:** 2026-06-26

**Authors:** Donghao Zhang, Xiaodan Wang, Na Pan, Wenxin Fan, Kunyan Sui

**Affiliations:** 1 College of Materials Science and Engineering, Key Laboratory of Marine Bio-Based Fibers of Shandong Province, Key Laboratory of Shandong Provincial Universities for Advanced Fibers and Composites, Qingdao University, Qingdao, 266071 People’s Republic of China; 2 College of Materials Science and Engineering, Liaocheng University, Liaocheng, 252000 People’s Republic of China

**Keywords:** Ionic skins, Electromagnetic interference shielding, Self-polarized potential, Self-orientation, Gradient polyelectrolyte hydrogel

## Abstract

Ionic skins (I-skins) integrated with high-performance electromagnetic interference (EMI) shielding, self-powering functionality, and sensitive pressure detection are critically needed for soft electronics, yet remains a considerable challenge. Herein, we report a facile diffusion-complexation strategy for fabricating self-oriented gradient MXene/polyelectrolyte hydrogels, which function effectively as self-powered I-skins with outstanding EMI shielding and pressure-sensing capabilities. A pivotal feature of our method is the concurrent coupling of two processes: osmotic pressure gradient- induced in-plane self-orientation of MXene nanosheets and diffusion-complexation reaction-induced spontaneous formation of a longitudinal charge gradient. This charge gradient endows the I-skins with a pressure-sensitive self-polarized potential, while the in-plane aligned MXene network imparts both exceptional EMI shielding performance and high-pressure sensitivity. Consequently, the resulting I-skins exhibit a high EMI shielding effectiveness of 48 dB, along with outstanding self-powered sensing capability that delivers a high sensitivity (3.067 mV kPa^−1^), a broad sensing range (0.05–80 kPa), a fast response time (120–130 ms), and a low detection limit for pressure (0.05 kPa). This work offers a new and scalable strategy for integrating high sensitivity, energy autonomy, and superior EMI shielding into advanced I-skins for broader applications.

**Supplementary Information:**

The online version contains supplementary material available at 10.1007/s40820-026-02255-z.

## Highlights

- The diffusion-complexation strategy enabled the synchronous construction of both an in-plane oriented MXene network and a longitudinal charge gradient structure within the hydrogel.
- An advanced ionic skin with an integrated architecture has been developed, which exhibits exceptional electromagnetic interference shielding performance along with outstanding self-powered sensing capability.

**Supplementary Information:**

The online version contains supplementary material available at 10.1007/s40820-026-02255-z.

## Graphical abstract

**Figure:**
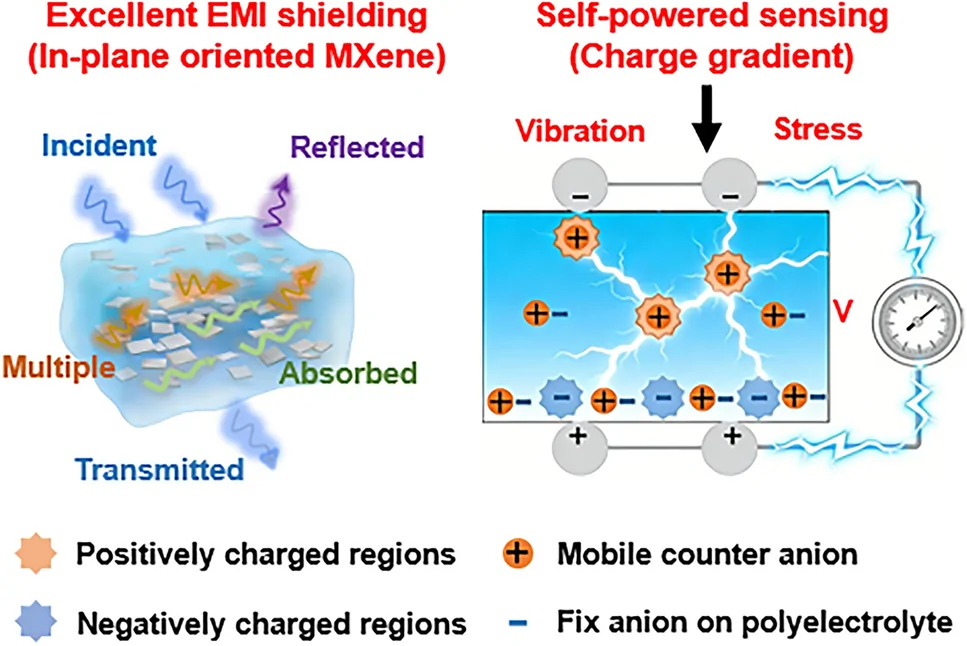

## Introduction

Ionic skins (I-skins) have garnered significant research interest owing to their inherent advantages in biomimetic signal transmission and mechanical compliance [1–4], which are crucial for emulating the sophisticated multifunctionality of natural human skin [5–9]. Advances in ionic conductors and microstructural engineering have enabled current I-skins to integrate a suite of bioinspired properties—such as self-healing [9–12], ultrastretchability [4, 5, 13, 14], ion gating [15–17], and damping [18]—with excellent sensing capabilities, including high sensitivity, low detection limits, and rapid response times [4, 12, 19–21]. Furthermore, self-powered operation has been achieved by introducing an asymmetric structure to generate a mechanical- and thermo-sensitive self-polarized potential along the thickness direction of I-skins [22–26], allowing efficient conversion of mechanical energy into electrical energy. This eliminates the need for bulky external power supplies and facilitates the development of energy-efficient, miniaturized, and lightweight devices.

However, the rapid evolution of high-speed wireless technologies (e.g., 5G and emerging 6G) and the proliferation of electronic devices have led to increasing electromagnetic pollution [27–34], which not only interferes with electronic systems, but also raises public health concerns [35–41]. This makes it imperative to incorporate effective electromagnetic interference (EMI) shielding into advanced I-skins [42–44]. To this end, several EMI-shielding I-skins (EMI-I-skins) have been developed by incorporating conductive fillers such as MXene or graphene oxide [45–48]. A key limitation is the need to keep conductive filler concentration low to preserve the intrinsic flexibility, biocompatibility, and ion-mediated signal transmission of I-skins [49], which inevitably compromises shielding effectiveness.

In this regard, constructing in-plane aligned structures with such two-dimensional (2D) conductive fillers has been demonstrated as an effective strategy for efficient electromagnetic wave absorption in electronic-based EMI materials [50–54], as the aligned network promotes multiple internal reflections and interfacial polarization, thereby enhancing EMI shielding performance [55–60]. Hence, introducing in-plane oriented architectures with 2D conductive fillers is a promising approach to achieve high EMI shielding performance under the constraint of low filler loading within I-skins. Nevertheless, a significant challenge lies in integrating this in-plane oriented network, essential for high EMI shielding performance, with a through-thickness charge gradient structure required for desired self-powered sensing capabilities. These two architectures are spatially orthogonal and typically require distinct, often conflicting, fabrication conditions. Therefore, the simultaneous integration of excellent EMI shielding performance and self-powering capability in a single I-skin system through a simple and scalable method remains a core scientific and technological hurdle.

**Fig. 1:**
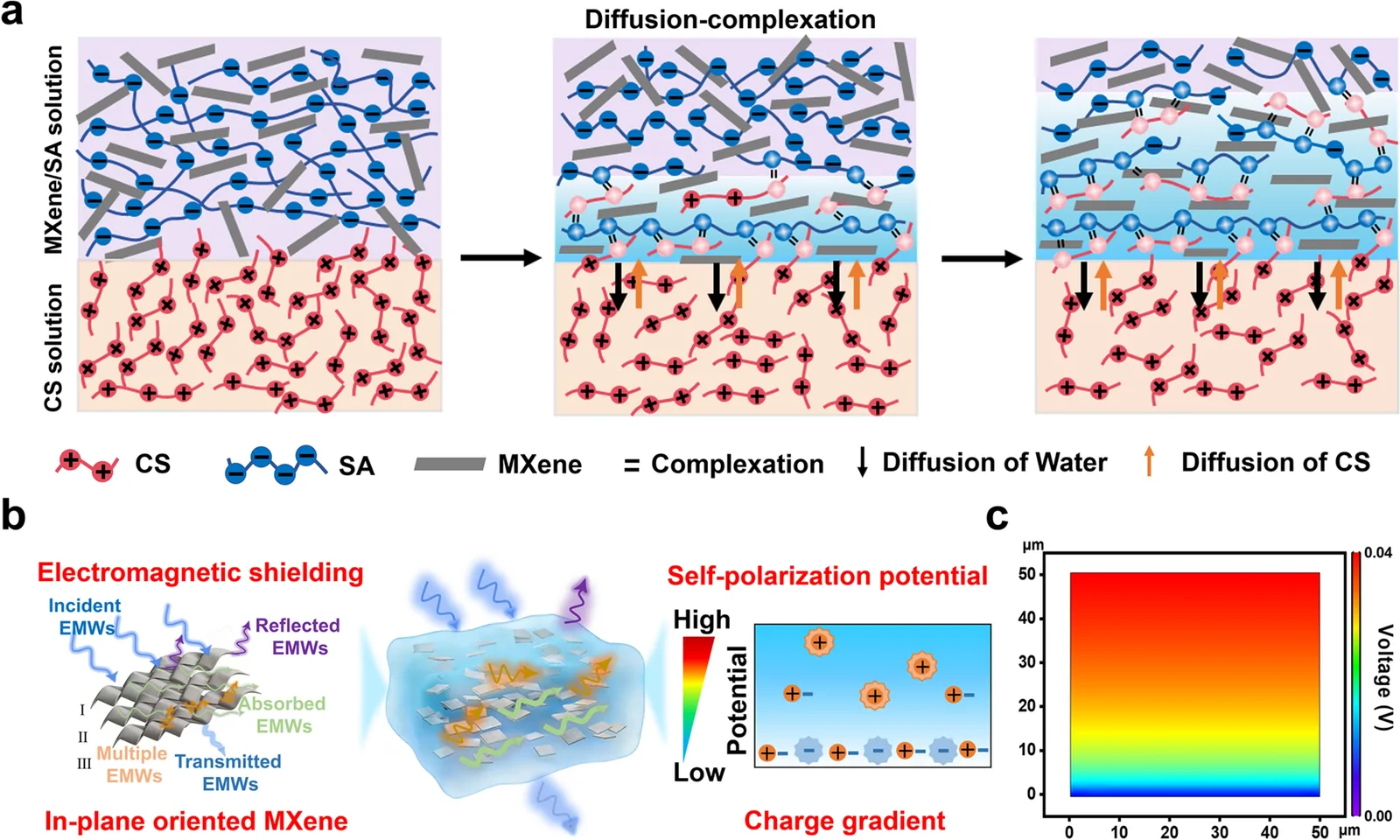
**a** Formation mechanism of gradient in MXene-SA/CS hydrogel films. **b** EMI shielding mechanism, and formation mechanism of self-polarized potential of self-oriented gradient MXene-SA/CS hydrogel film. **c** Simulated potential profiles within the self-oriented gradient MXene-SA/CS hydrogel film (See Notes S2, S3)

To overcome these challenges, we propose a novel diffusion-complexation approach to simultaneously create an in-plane oriented MXene structure and a longitudinal charge gradient within I-skins, thereby endowing the material with integrated superior EMI shielding performance, high sensing performance, and self-powering capability (Fig. 1). This strategy is realized through a one-step immersion process, where a concentrated solution of low molecular weight (LMW) polycations is brought into contact with a mixture solution of high molecular weight (HMW) polyanions and MXene. The concentrated LMW polycation solution serves two essential roles: (i) as a reactant that diffuses into the HMW polyanion/MXene mixture solution and complexes electrostatically with the polyanions, forming a gradient polyelectrolyte hydrogel matrix; and (ii) as an osmotic pressure regulator whose high osmotic potential induces directional deswelling and compression of the matrix along the thickness direction, thereby driving the in-plane self-alignment of MXene nanosheets (Fig. 1a). This charge gradient endows the I-skins with a pressure-sensitive self-polarized potential, while the in-plane aligned MXene network imparts both exceptional EMI shielding performance and high-pressure sensitivity (Fig. 1b, c). Consequently, the resulting I-skins achieve a high EMI shielding effectiveness of 48 dB, along with a self-powered sensing capability that delivers high sensitivity (3.067 mV kPa^−1^), a wide sensing range (0.05–80 kPa), a fast response speed (120–130 ms), and a low-pressure detection limit (50 Pa). This straightforward and scalable methodology offers new perspectives for integrating outstanding EMI shielding performance with high sensing performance and diverse bioinspired properties within the advanced I-skins.

## Experimental Section

### Material Synthesis

#### Materials

Lithium fluoride (LiF) was purchased from Shanghai McLean Biochemical Co., Ltd. Sodium alginate (SA) was purchased from Qingdao Hyzlin Biology Development Co., Ltd. LMW chitosan (CS) was purchased from Weifang Dongxing Crustacean Products Factory. Hydrochloric acid (HCl) was purchased from Aladdin Reagent. Ternary carbide precursor titanium aluminum carbide (MAX Ti_3_AlC_2_, 200 mesh) powder was purchased from 11 Technology. All reagents were used directly without further purification.

#### Synthesis of MXene

The Ti_3_C_2_T_x_ MXene was synthesized via a chemical etching approach. The detailed procedure is described as follows. First, 1.6 g of LiF was added to 40 mL of 9 M HCl in a polytetrafluoroethylene vessel under continuous stirring for 10 min to achieve complete mixing. Subsequently, 1 g of Ti_3_AlC_2_ powder (with an average particle size below 38 μm) was introduced into the mixture, followed by continuous stirring at 37 °C for 24 h to ensure thorough etching. The resulting product was repeatedly washed with deionized water until the supernatant reached a pH value above 5, thereby removing residual acidic components. The etched material was then subjected to ultrasonication for 30 min to exfoliate the MXene layers, followed by centrifugation to separate and discard impurities. Finally, layered Ti_3_C_2_T_x_ MXene suspension was obtained. This method allowed us to obtain high-quality MXene materials.

#### Preparation of Self-Oriented Gradient MXene-SA/CS Hydrogels

Self-oriented gradient MXene-SA/CS hydrogels were fabricated via a reaction–diffusion approach. A homogeneous MXene/SA mixture solution was first prepared by combining 0.25–1.5 wt% sodium alginate (SA) with 0.25–2.0 wt% MXene powder, followed by 30 min of ultrasonic treatment. Subsequently, a culture dish containing 50 wt% low-molecular-weight chitosan (LMW CS) solution was immersed into the MXene/SA mixture. During this process, LMW CS diffused into the MXene/SA mixture and crosslinked with SA through electrostatic interactions, resulting in the formation of a gradient MXene-SA/CS hydrogel film at the interface between the two solutions. The high concentration of LMW CS further induced directional deswelling of the hydrogel film along the thickness direction, promoting in-plane self-orientation of both MXene nanosheets and the SA/CS polymer network. After a reaction period of 10–60 min, the self-oriented gradient MXene-SA/CS hydrogel film was carefully retrieved from the solution interface. Any unreacted residues on the surface were removed by rinsing with distilled water.

#### Construction of Self-Powered I-Skin

The thickness-dependent and interface-dependent self-powered I-skins were constructed by sandwiching the self-oriented gradient MXene-SA/CS hydrogels between two Pt electrodes and two silver mesh electrodes, respectively. The pressure-sensing performance tests were all conducted using a 1 cm^2^ hydrogel with a thickness of approximately 1 mm at a room temperature of 25 °C.

### Characterization

The cross-sectional microstructure of the samples was characterized using a field emission scanning electron microscope (FE-SEM, JSM-7800, Hitachi, Japan). The as-prepared SA/MXene/CS hydrogels were rapidly frozen to induce brittle failure in liquid nitrogen and subsequently freeze-dried to preserve their internal architecture. The dried specimens were mounted onto conductive adhesive tape, sputter-coated with a thin layer of gold, and examined under an accelerating voltage of 10 kV to reveal the morphological features of the samples. The microstructure of MXene was characterized using a JEM-2100 transmission electron microscope (TEM) operated at an accelerating voltage of 200 kV. For sample preparation, a diluted MXene dispersion was drop-cast onto a carbon-film-coated copper grid and allowed to air-dry naturally prior to imaging. The electromagnetic interference (EMI) shielding performance of the samples was evaluated in the X-band frequency range (8.2–12.4 GHz) using an E5063A vector network analyzer (VNA, American Technology). Specimens were prepared by cutting the materials into rectangular shapes measuring 20 mm × 30 mm for testing. Fourier transform infrared (FTIR) spectroscopy (Nicolet 5700, Thermal Electron Corporation, USA) was employed to characterize the chemical structures of SA, CS, MXene, and the composite MXene-SA/CS hydrogels, with spectra collected over a wavenumber range of 400 to 4000 cm^−1^. X-ray diffraction (XRD) analysis was performed using a DX2700 diffractometer equipped with a Cu K*α* radiation source to examine the crystalline structure of the samples. The measurements were conducted at 40 kV and 30 mA, with a scanning angle (2*θ*) ranging from 5° to 80° at a rate of 0.03° s^−1^. The conductivity of the samples (25–30 mm in diameter and 3–4 mm in thickness) was measured with an RTS-8 four-probe resistivity meter. The sensing properties were tested by a digital source meter (Keithley 2450). The mechanical properties of the samples were characterized using a testing machine (WDW-5 T) under a span of 10 mm at a tensile speed of 20 mm min^−1^.

### Theoretical Calculation of EMI Shielding

EMI shielding effectiveness is defined as the logarithmic ratio of incident power to transmitted power. The total SE (SE_T_) is the sum of SE_R_, SE_A_, and SE_M_. SE_M_ can be neglected for SE greater than 15 dB. The scattering parameters (S11, S21, and S22) were measured to obtain SE_T_, SE_A_ and SE_R_ according to the formula:

1 $${\mathrm{SE}}_{T} {\text{ = 10log}}\left( {\frac{1}{{\left| {S_{21} } \right|^{2} }}} \right)$$

2 $${\mathrm{SE}}_{R} = 10{\mathrm{log}}\left( {\frac{1}{{1 - \left| {S_{11} } \right|^{2} }}} \right)$$

3 $${\mathrm{SE}}_{A} = 10{\mathrm{log}}\left( {\frac{{1 - \left| {S_{11} } \right|^{2} }}{{\left| {S_{21} } \right|^{2} }}} \right)$$

The absorption coefficient (*A*), reflection coefficient (*R*) and transmission coefficient (*T*) are calculated as follows:

4 $$T = \left| {S_{12} } \right|^{2} = \left| {S_{21} } \right|^{2}$$

5 $$R = \left| {S_{11} } \right|^{2} = \left| {S_{22} } \right|^{2}$$

6 A+R+T=1

## Results and Discussion

### Self-Oriented Gradient MXene/Polyelectrolyte Hydrogel

For the proof of concept, the LMW chitosan and HMW sodium alginate were employed as the polycation and polyanion, respectively, in the construction of the hydrogel matrix. The Ti_3_C_2_T_x_ MXene nanosheets utilized in this study exhibit an average lateral dimension of approximately 0.2 μm and an average thickness of 1.6 μm (Fig. S1). Detailed synthesis procedures and characterization of the MXene nanosheets are provided in the experimental section and Fig. S2. Figure 1 illustrates the principle of the diffusion-complexation method used to concurrently fabricate a laminated self-oriented MXene structure and a charge gradient within the MXene-SA/CS hydrogel films. Typically, MXene nanosheets were first dispersed in a 1 wt% HMW SA solution to form the homogeneous MXene/SA mixture. Subsequently, by immersing a culture dish containing 50 wt% LMW CS solution into the MXene/SA mixture, a robust self-oriented gradient MXene-SA/CS hydrogel film formed directly at the interface of the two solutions within minutes. Here, the highly concentrated LMW CS solution fulfills two critical functions. First, it acts as a reactant that diffuses into the MXene/HMW SA mixture and complexes with SA via electrostatic interactions, thereby generating MXene-SA/CS hydrogel layers with a charge gradient at the interface. The bottom side adjacent to the LMW CS solution (denoted as the LD side) exhibits a lower density of residual negative charges compared to the opposite side (denoted as the HD side), since the SA at bottom side could react with more CS [23]. Second, the highly concentrated LMW CS solution serves as an osmotic pressure regulator. Its high osmotic pressure induces deswelling and compression of the polyelectrolyte matrix along the thickness direction, leading to in-plane self-orientation of the MXene nanosheets. The osmotic pressure of LMW CS solution is as high as 4.75 MPa, much higher than the 0.6 MPa of MXene/SA mixture solution (Fig. S3). This result demonstrates the existence of a significant osmotic pressure gradient along the thickness of the resulting MXene-SA/CS hydrogel. Additionally, due to the high osmotic pressure gradient, the LD side loses more water and exhibits a higher content of SA/CS polymer networks and MXene nanosheets relative to the HD side. Throughout this process, LMW CS continuously complexes with SA, thereby stabilizing the oriented structure as well as the MXene-SA/CS network density gradient and charge gradient. As a result, the as-prepared MXene-SA/CS hydrogel film features both an in-plane self-aligned MXene nanostructure and a gradient architecture, characterized by lower negative charge density and lower water content on the LD side compared to the HD side. The thickness of the MXene-SA/CS hydrogel film is tunable over a broad range, from 400 to 750 μm, by varying the diffusion time between 10 and 60 min (Fig. S4).

**Fig. 2:**
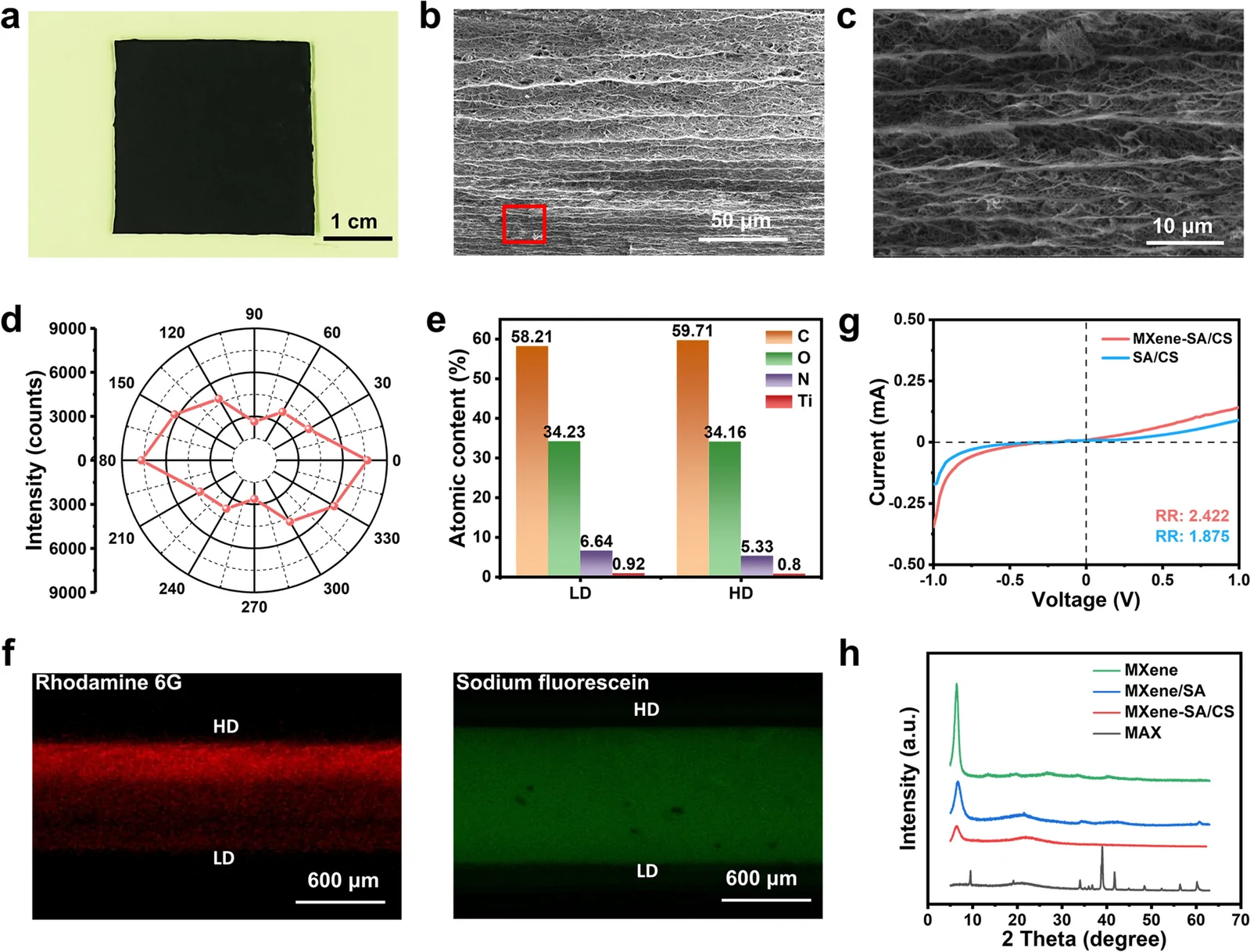
**a** Optical image of self-oriented gradient MXene-SA/CS hydrogel. **b** Cross-sectional SEM images of MXene-SA/CS hydrogel and **c** the partial enlarged view. **d** Polarized Raman images of MXene-SA/CS hydrogel. **e** Atomic percentage of C, O, N and Ti on the HD and LD sides of gradient MXene-SA/CS hydrogel taken from XPS spectra. **f** CLSM images of MXene-SA/CS hydrogel film dyed by the rhodamine 6G and sodium fluorescein. **g** I-V curve of MXene-SA/CS hydrogel and SA/CS hydrogel under a voltage of -1 to 1 V. **h** XRD images of MXene, MXene/SA, MXene-SA/CS and MAX

Figure 2a shows the optical image of a self-aligned gradient MXene-SA/CS hydrogel membrane, fabricated using a mixture of 1.0 wt% MXene/1.0 wt% SA and 50 wt% CS solution. The scanning electron microscope (SEM) images of cross sections at different magnifications reveals the presence of in-plane aligned layered structures within the hydrogel film, indicating the preferential alignment of MXene nanosheets along the planar direction (Fig. 2b, c). In contrast, no such layered morphology is observed in the cross-sectional SEM image of the SA/CS hydrogel film prepared without MXene (Fig. S5). The in-plane self-orientation of MXene nanosheets was further corroborated by polarized Raman spectroscopy (Fig. 2d). The 150–200 cm^−1^ region corresponds to the out-of-plane vibration of Ti atoms in MXene, which is highly sensitive to the in-plane polarization angle, with the intensity exhibiting periodic variations with the angle (Fig. S6). By normalizing the peak intensity at 150 cm^−1^ under different polarization angles and plotting it into a polar coordinate diagram, the shape of the graph significantly deviates from a circle, indicating a well-defined orientation of MXene within the sample (Fig. 2d) [61]. The Herman orientation parameter of the self-orienting gradient MXene-SA/CS hydrogel film was calculated to be 0.0835 (see more details in Note S1). Furthermore, we characterized the orientation of the polymer networks in the SA/CS hydrogel matrix without MXene nanosheets using polarizing optical microscopy (POM, Fig. S7). The distinct optical anisotropy observed under polarized light (i.e., dark field view perpendicular to the plane and bright interference colors parallel to the plane) confirms that the SA/CS polymer networks are oriented along the in-plane direction (Fig. S7). Additionally, as anticipated, the MXene-SA/CS hydrogel film exhibits a denser MXene-SA/CS network at the LD side relative to the HD side (Fig. 2b, c). To confirm the distribution of MXene, Raman spectra were collected from different positions of the MXene-SA/CS hydrogel, including the top (HD) side, the middle region, and the bottom (LD) side (Fig. S8). The peak near 150–200 cm^−1^ corresponds to the in-plane vibration of the Ti-C bond in MXene, which is one of the most representative characteristic peaks of MXene [62]. The results showed that the MXene/Ti content gradually increased from the HD side to the LD side, attributed to the significant deswelling of the LD side induced by the osmotic pressure gradient (Fig. S8). Furthermore, the surface atomic percentages of carbon, nitrogen, oxygen, and titanium on the HD and LD sides were analyzed using X-ray photoelectron spectroscopy (XPS, Figs. 2e and S9). A higher nitrogen content at the LD side manifests a higher concentration of LMW CS at this side. The molar ratios of –COONa (from SA) to –NH_2_ (from CS) at the LD and HD sides are estimated to be 0.1 and 0.5, respectively (Fig. S10), indicating a lower negative charge density at the LD side compared to the HD side. This charge gradient along the thickness direction of the MXene-SA/CS hydrogel is visually confirmed by confocal laser scanning microscopy (CLSM) images (Fig. 2f), which show a gradient distribution of positively charged rhodamine 6G within the film, while sodium fluorescein does not exhibit a pronounced gradient [25]. The asymmetric charge distribution enables both the gradient MXene-SA/CS hydrogel and gradient SA/CS control sample to rectify the ion current, achieving the rectification ratios of approximately 2.422 and 1.875 for the MXene-SA/CS hydrogel and SA/CS control sample, respectively (Fig. 2g). The analysis of MXene's different states via X-ray diffraction (XRD) revealed that the characteristic peak (002) showed no significant shift across states and no new peaks formed (Fig. 2h). This indicates MXene exhibits excellent dispersibility in both solution and gel states without noticeable aggregation (Fig. 2h). Furthermore, we have characterized the viscosity of MXene/SA precursor solutions with varying MXene concentrations (Fig. S11). As the MXene content increased, the viscosity initially rose due to extensive hydrogen bonding interactions between SA and MXene, reaching a peak at 1.25 wt% MXene. However, further increasing the MXene content to 1.5 wt% led to a decrease in viscosity, because of reduced hydrogen bonding interactions caused by MXene aggregation. These results also confirm that MXene exhibits excellent dispersibility in the MXene/SA precursor solution at concentrations below 1.25 wt%.

### EMI Shielding Performance

To illustrate the process of EMI shielding, a simplified schematic is presented in Fig. 1b. When electromagnetic waves encounter the shielding material, a portion of the waves are reflected off the surface due to impedance mismatch and the porous nature of the hydrogel film. The remaining waves undergo a series of reflections and scattering events within the layered structure of the film. The MXene material within the gel is arranged in an orderly manner, and its high electrical conductivity and abundance of free electrons result in the rapid reflection of some electromagnetic waves that encounter its surface. Those waves that penetrate the MXene lattice structure interact with its high electron density, generating a current and causing energy loss. Waves passing through the first layer of Ti_3_C_2_T_x_ (labeled “I” in Fig. 1b) continue to meet subsequent barrier layers (labeled “II”), repeating the attenuation process. Each layer serves as a reflective interface, initiating multiple internal reflections. Reflection within the layers, interference between the layers, and the high electron density of MXene together enhance the full absorption of electromagnetic waves within the material. This layered structure endows the MXene with a multi-layered shielding effect. In summary, the shielding effectiveness of the MXene-SA/CS composite film is augmented by the combined effects of reflective, absorptive, as well as multiple internal reflections for prolonging the propagation path of microwaves within the hydrogel and further enhancing its absorption capacity.

**Fig. 3:**
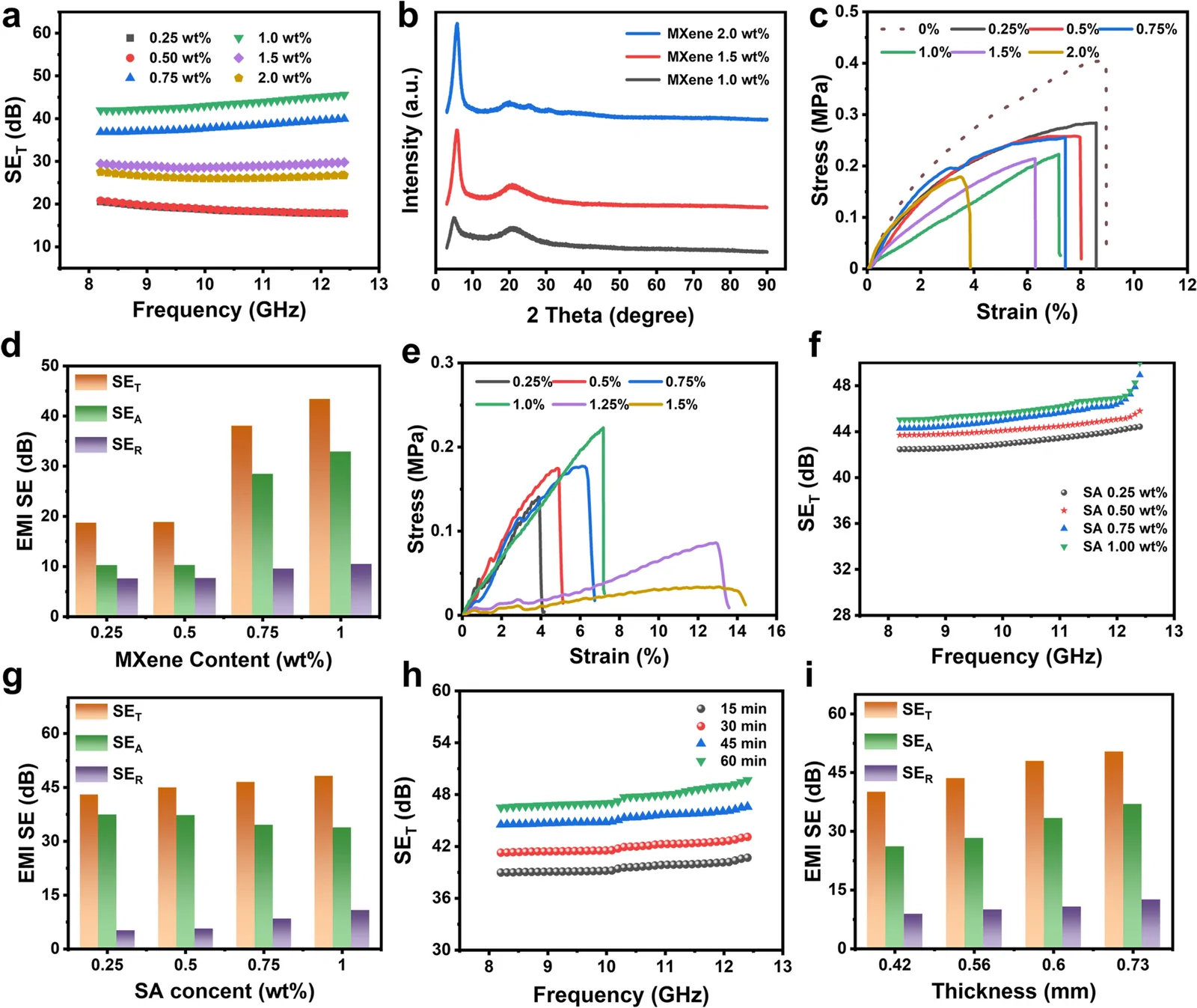
**a** EMI shielding properties, **b** XRD curves, **c** stress–strain curves, and **d** SE_T_, SE_A_ and SE_R_ of MXene-SA/CS hydrogels prepared with different MXene contents. **e** Stress–strain curves, **f** EMI shielding properties, and SE_T_, **g** SE_A_ and SE_R_ of MXene-SA/CS hydrogels prepared with different SA contents. **h** EMI shielding properties, and **i** SE_T_, SE_A_ and SE_R_ of MXene-SA/CS hydrogels with different thickness

Electrical conductivity is a critical factor governing the performance of electromagnetic interference (EMI) shielding materials. As depicted in Fig. S12, the electrical conductivity of MXene-SA/CS hydrogels was evaluated as a function of MXene content. The conductivity exhibits a positive correlation with the MXene concentration within the range of 0.25–1 wt%, which is attributed to the formation of more extensive conductive networks. When the concentration of MXene increases over 1 wt%, the conductivity decreases with the MXene concentration increasing, due to the restacking and aggregation of nanosheets. The XRD curves also reveal the decrease in interlayer spacing and the formation of MXene nanoplate aggregates with increasing the MXene concentration, as reflected by the shift of characteristic peak for the (002) crystal plane to the larger *θ* (Fig. 3b). It is worth noting that besides the MXene-SA/CS hydrogels prepared with the ultralow MXene contents (≥ 0.25 wt%), the overall conductivities of other MXene-SA/CS hydrogels remain within the range of 0.4–0.45 S m^−1^, demonstrating their excellent electrical conductivity.

The X-band EMI shielding performance of MXene-SA/CS hydrogels was investigated using a vector network analyzer. The EMI shielding effectiveness (SE) values for MXene-SA/CS hydrogels with varying MXene and SA contents and thicknesses are presented in Fig. 3. As anticipated, the total EMI shielding effectiveness (SE_T_) first increased and then decreased with increasing MXene content (Fig. 3a). The SE_T_ of MXene-SA/CS hydrogel can reach to a maximum value of 45.4 dB when the 1 wt% MXene is used, exceeding the commercial requirement for EMI shielding materials (20 dB). Total EMI shielding effectiveness (SE_T_) comprises absorption effectiveness (SE_A_), reflection effectiveness (SE_R_), and multiple internal reflection effectiveness (SE_M_), and the relationship among the three is detailed in Eqs. (1–6). Clearly, SE_A_ dominated SE_T_ for all samples (Fig. 3d). When SE_T_ exceeds 15 dB, SE_M_ is typically negligible. With the increase in MXene content, SE_A_ increased from 10 to 33 dB, while SE_R_ remained almost unchanged, the MXene-SA/CS hydrogel exhibited excellent EMI performance. The excellent conductivity of the conductive filler MXene, combined with the gradient layered structure of the film, contributes to the EMI shielding effectiveness of MXene-SA/CS hydrogel. As the concentration of MXene increases, the SE_A_ gradually rises and is much higher than that of the SE_R_, indicating an increase in conductivity and a corresponding increase in the consumption of electromagnetic waves within the hydrogel (Fig. 1b). The mechanical properties of MXene-SA/CS hydrogels with varying MXene contents were also investigated (Fig. 3c), since the good mechanical properties are also critical for the practical application. Both the tensile strength and elongation at break of MXene-SA/CS hydrogels gradually decrease with increasing MXene content, implying the introducing of the MXene degrades the electrostatic complexation density between the SA and CS. Considering both the EMI performance and mechanical properties, the MXene-SA/CS hydrogel prepared with 1 wt% MXene was used.

Similarly, we also optimized the SA content according to the mechanical properties and ESI performance. As shown in Fig. 3e, the tensile strength of the MXene-SA/CS hydrogels first increases and then decreases sharply as SA content rises from 0.25 to 1.5 wt%. Optimal mechanical performance was achieved at 1.0 wt% SA. This is because the low SA concentration causes the limited electrostatic complexation density, while excessively high SA concentration leads to formation of ultradense CS/SA complex membrane, which only allows a small amount of CS to diffuse across it for further reaction [23]. Given the mechanical properties, we employed SA concentrations between 0.25 and 1.0 wt% for subsequent EMI shielding performance testing. Overall, the contribution of SA content to EMI shielding is not sufficiently pronounced (Fig. 3f, g). As the SA content increases from 0.25 to 1.0 wt%, the SE_T_ rises slightly from 43.5 to 48.6 dB (Fig. 3f), among which the SE_A_ decreases from 37.4 to 33.8 dB, and the SE_R_ increases from 5.1 to 10.8 dB (Fig. 3g). Here, the decrease in SE_A_ stems from the denser polymer networks and the decreased pore size of the MXene-SA/CS hydrogel (i.e., shorter propagation path of microwaves), as shown in Figs. 2b and S13. The 1 wt% SA was used in the following test because of both high EMI and mechanical performances of corresponding MXene-SA/CS hydrogels.

Concurrently, sample thickness exerts a highly significant influence on SE_T_. Figure 3h, i illustrates the SE_T_ performance of hydrogels prepared with 1.0 wt% MXene and 1.0 wt% SA. As hydrogel thickness increases from 0.42 to 0.73 mm, SE_T_ enhances from 40 to 50 dB. The increased thickness provides more opportunities for penetrating electromagnetic waves to interact with conductive pore walls, and the waves undergo multiple reflections at the air/pore wall interface, dissipating their energy. Therefore, subsequent experiments primarily explored the application of MXene-SA/CS hydrogels with a thickness of approximately 0.7 mm, prepared with 1.0 wt% MXene and 1.0 wt% SA.

We also evaluated the long-term stability of the EMI shielding performance of the MXene-SA/CS hydrogels. Prior to testing, we encapsulated the hydrogel with commercial polydimethylsiloxane (PDMS) membranes to prevent water evaporation. The MXene-SA/CS hydrogels maintained good EMI shielding performance, with only a negligible decrease of 3.8% in SE_T_ after ambient storage over three weeks (Fig. S14). In this work, the gradient structure has little influence on the EMI performance. When electromagnetic waves were incident from the HD side and the LD side, the EMI shielding effectiveness was 47.6 and 48.2 dB, respectively, indicating little overall difference (Fig. S15). In both cases, the EMI shielding of the I-skin is dominated by absorption. Notably, the material exhibits relatively stronger absorption and weaker reflection of electromagnetic waves when they are incident from the HD side. This is because the electromagnetic waves entering from the HD side are not strongly reflected at the surface; instead, they penetrate more readily into the interior, leading to more extensive multiple reflections and energy dissipation.

### Self-Powered Sensing Performance

Owing to the diffusion of counter ions under the concentration gradient, there exists a self-polarized transmembrane potential (*V*_*s*_) across the self-oriented gradient MXene-SA/CS hydrogel films. The self-polarized transmembrane potential (*V*_*s*_) can be represented as [63]:

7 $$V_{s} { = }\frac{RT}{{z_{i} F}}{\mathrm{ln}}\frac{{c_{i} \left( h \right)}}{{c_{i} \left( l \right)}}$$

where *c*_*i*_(h) and *c*_*i*_(l) are the concentrations of i at the high charge density (HD) side and at low charge density (LD) side of gradient hydrogel, respectively.

The I-skins can be constructed by sandwiching the MXene-SA/CS hydrogel film between two electrodes. The relationship between the output voltage (*V*_0_) and self-polarized transmembrane potential (*V*_*s*_) can be represented as:

8 $$V_{0} { = }V_{s} { - 2}V_{i} { = }V_{s} { - 2}Q/C_{i}$$

where *Q* represents the charge quantity at the electrode/ion diode interface, *V*_*i*_ and *C*_*i*_ denote the voltage drop and capacitance at the electrode/hydrogel interface, respectively.

**Fig. 4:**
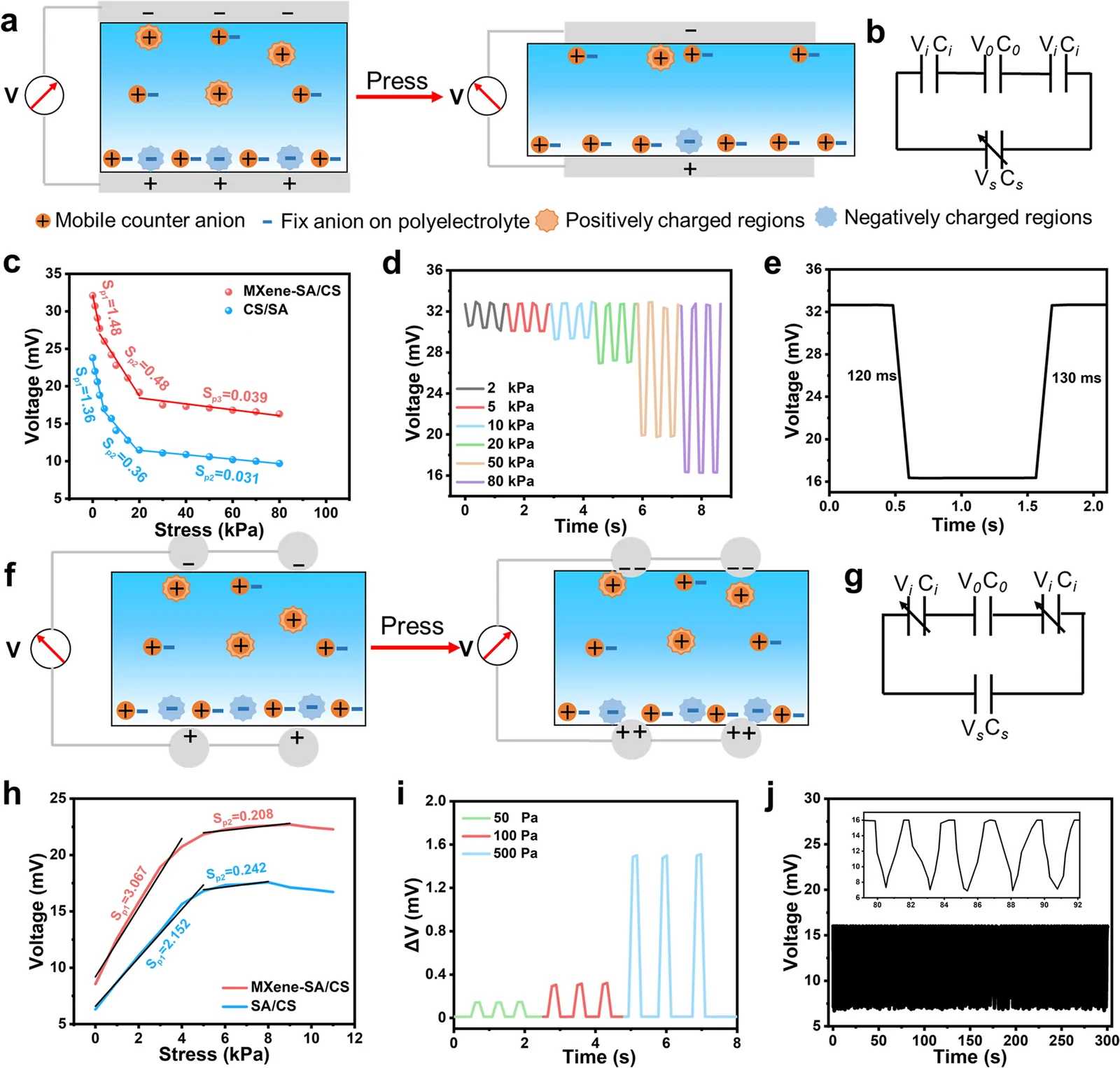
**a** Working mechanism and **b** equivalent circuit of the thickness-dependent self-powered I-skins. **c** Compression sensitivity of thickness-dependent self-powered I-skins. **d** Open-circuit voltage of thickness-dependent self-powered I-skins in response to the different pressures. **e** Response/recovery time of the thickness-dependent self-powered I-skins in response to 80 kPa pressure. **f** Working mechanism and **g** equivalent circuit of the interface-dependent self-powered I-skins. **h** Output voltage of the interface-dependent self-powered I-skins constructed with the gradient MXene-SA/CS hydrogel and the SA/CS control hydrogel, in response to gradually increasing pressure. Open-circuit voltage of interface-dependent self-powered I-skins in response to **i** the different pressures and **j** the repeated loading and unloading of 2 kPa pressure for 200 cycles

Here, two types of self-powered I-skins, including thickness-dependent and interface-dependent self-powered I-skins, were constructed by using smooth and rough electrodes, respectively (Fig. 4). Specifically, when two smooth platinum (Pt) electrodes were used, the interface area and capacitance (*C*_*i*_) between the I-skins and electrodes are constant under the pressure (Fig. 4a). In this case, the output voltage of I-skins is determined by the self-induced potential of gradient MXene-SA/CS hydrogel films (Eq. 8) [25]. The gradient MXene-SA/CS hydrogel films can be simplified as an aggregation of numerous ultrathin homogeneous layers with identical thickness, and the density of net charged groups of ultrathin layers increases gradually along the gradient/thickness direction [64]. When a pressure is applied on the I-skins, each ultrathin layer within gradient hydrogel film thinner, and the diffusion distance of counterions is shortened. Hence, more counterions (i.e., Na^+^) diffuse from the top layer to the bottom layer, and the corresponding value of *c*_*i*_(h)/*c*_*i*_(l) decreases (Fig. S16), leading to the decrease in the self-polarized potential of gradient hydrogel films and output voltage of I-skins (Fig. 4b and Eqs. 7 and 8). Therefore, the resulting self-powered I-skin operates in a thickness-dependent mode in response to pressure change. We also constructed the such thickness-dependent self-powered I-skins using the gradient SA/CS hydrogel film, and compared the maximum output voltage of our MXene-SA/CS hydrogel-based and gradient SA/CS hydrogel-based I-skins under the pressure-free state (Fig. 4c). Intriguingly, the incorporation of MXene nanosheets significantly enhanced the output voltage to ~ 32.1 mV, compared to only ~ 24.8 mV for the MXene-free SA/CS hydrogel. This enhancement is attributed to the surface-located MXene nanosheets, which function as extended electrode interfaces. These interfaces substantially increase the effective electrode-hydrogel contact area, thereby amplifying the electrical double-layer capacitance (*C*_*i*_), and increasing the output voltage (Eq. 8). The pressure sensitivity, defined as *S* =|Δ*V*/Δ*P*|, is used to quantificationally evaluate the sensing performance of self-powered I-skins, where Δ*V* and Δ*P* represent changes in output voltage and pressure, respectively. The pressure sensitivities of MXene-SA/CS hydrogel-based I-skin are 1.48, 0.48, and 0.039 mV kPa^−1^, in the pressure ranges of 2–3, 3–20, and 20–80 kPa, respectively. In contrast, the pressure sensitivities of gradient SA/CS hydrogel-based I-skin are only 1.36, 0.36, and 0.031 mV kPa^−1^, in the pressure ranges of 2–5, 5–20, and 20–80 kPa, respectively. This is because a high output voltage of self-powered I-skins is beneficial for a high-pressure sensitivity (Fig. S17, See more detailed description in Note S4) [65]. The self-powered MXene-SA/CS hydrogel-based I-skins present reversible and stable electrical signal in response to cyclic loading/unloading of pressure in a large range (Fig. 4d). Meanwhile, the resulting self-powered I-skins hold high response speed, as reflected by an extremely short response time of 120 and 130 ms in response to the loading and unloading of an 80 kPa pressure, respectively (Fig. 4e). Besides, the self-powered I-skins hold excellent durability, and maintain a stable electrical response under repeated loading and unloading of 80 kPa pressure for more than 200 cycles (Fig. S18).

The MXene-SA/CS hydrogel-based self-powered I-skins can also work in the interface-dependent mode when the two rough silver mesh electrodes were used (Fig. 4f). When a small pressure was applied on the I-skins, the thickness of the gradient MXene-SA/CS hydrogel remains nearly unchanged, and hence its self-polarized potential presents a little change. In this case, the output voltage of I-skins is dominated by the interface capacitance between the gradient hydrogel and electrodes (Fig. 4g and Eq. 8). When a small pressure is applied onto the I-skin, the gradient hydrogel/electrode interface capacitance, directly proportional to interface area, greatly enlarges, leading to the sharp decrease in the interface potential loss (*V*_*i*_) and pronounced increase in the output voltage. Continuously increasing pressure causes the gradual increase in the output voltage. However, when the pressure increases over a threshold value, further increasing the pressure gives rise to the decrease in output voltage (Fig. 4h). This is attributed to the decrease in the self-polarized voltage of gradient hydrogels induced by the decrease in thickness [25].

For the interface-dependent gradient MXene-SA/CS hydrogel-based I-skins, the pressure can not only enlarge the interface area and interface capacitance between the gradient hydrogel films and electrodes, but also allow the electrodes to contact with more MXene, which can act extended electrode interfaces to further increase the interface capacitance. Therefore, when used as the interface-dependent self-powered I-skins, self-powered oriented gradient MXene-SA/CS hydrogel-based I-skin also presents much higher sensitivity than the gradient SA/CS hydrogel-based I-skin (Fig. 4h). The pressure sensitivities of resulting I-skin are as high as 3.067 and 0.208 mV kPa^−1^ in the pressure ranges of 0.05–5 and 5–10 kPa, respectively. The high output voltage of the gradient MXene-SA/CS hydrogel enables the corresponding interface-dependent self-powered I-skins to achieve high sensitivity, compared to that of gradient SA/CS hydrogel-based self-powered ionic skins (Fig. S17, Notes S4 and S5 for a more detailed description). The interface-dependent self-powered I-skins can precisely detect the extremely small pressure of 50 Pa, and exhibit high-stability response signals in response to 0.05–0.5 kPa pressure (Fig. 4i). Compared to other ion-based gradient hydrogels, ours exhibits higher sensing sensitivity (Fig. S19). Meanwhile, the resulting self-powered I-skins hold high response speed, as reflected by an extremely short response time of 120 and 120 ms in response to the loading and unloading of an 0.05 kPa pressure, respectively (Fig. S20). In addition, these interface-dependent self-powered I-skins demonstrate excellent durability and stability, maintaining a stable electrical response under repeated loading and unloading of a 2 kPa pressure for over 200 cycles (Fig. 4j). Notably, after encapsulation with a PDMS membrane, no significant output voltage drift is observed in the response curves during repeated loading and unloading of a 2 kPa pressure following three weeks of ambient storage in ambient air. These results confirm the outstanding long-term sensing stability of the self-powered I-skin (Fig. S21). Based on these two types of power supply devices, different human behaviors under varying pressures were clearly distinguished according to the stable potential shift across different force conditions (Fig. S22). This also provides robust support for the application of MXene-SA/CS hydrogel sensors in real-time monitoring of human activities.

To better position the contribution of this work within the field of multifunctional I-skins, we have provided a systematic comparison table (Tab. S1) that benchmarks key metrics—including SE_T_, sensitivity (mV kPa^−1^), filler loading, mechanical performance, and self-powering capacity—between our MXene-SA/CS gradient hydrogel-based self-powered I-skin and recently reported MXene-based hydrogels or gradient polyelectrolyte systems. Compared with recently reported gradient polyelectrolyte systems, our MXene-SA/CS gradient hydrogel-based self-powered I-skin not only exhibits higher sensitivity, but also delivers outstanding EMI shielding performance as an additional functionality. Furthermore, in comparison with previous electron-conducting MXene-based hydrogels, our self-powered I-skin, featuring an ultralow MXene content and the ion-based signal carriers, achieves comparable or even higher SE_T_ while additionally offering excellent self-powered sensing capability (Tab. S1).

### Applications of MXene-SA/CS Hydrogel-Based Self-Powered I-Skins

**Fig. 5:**
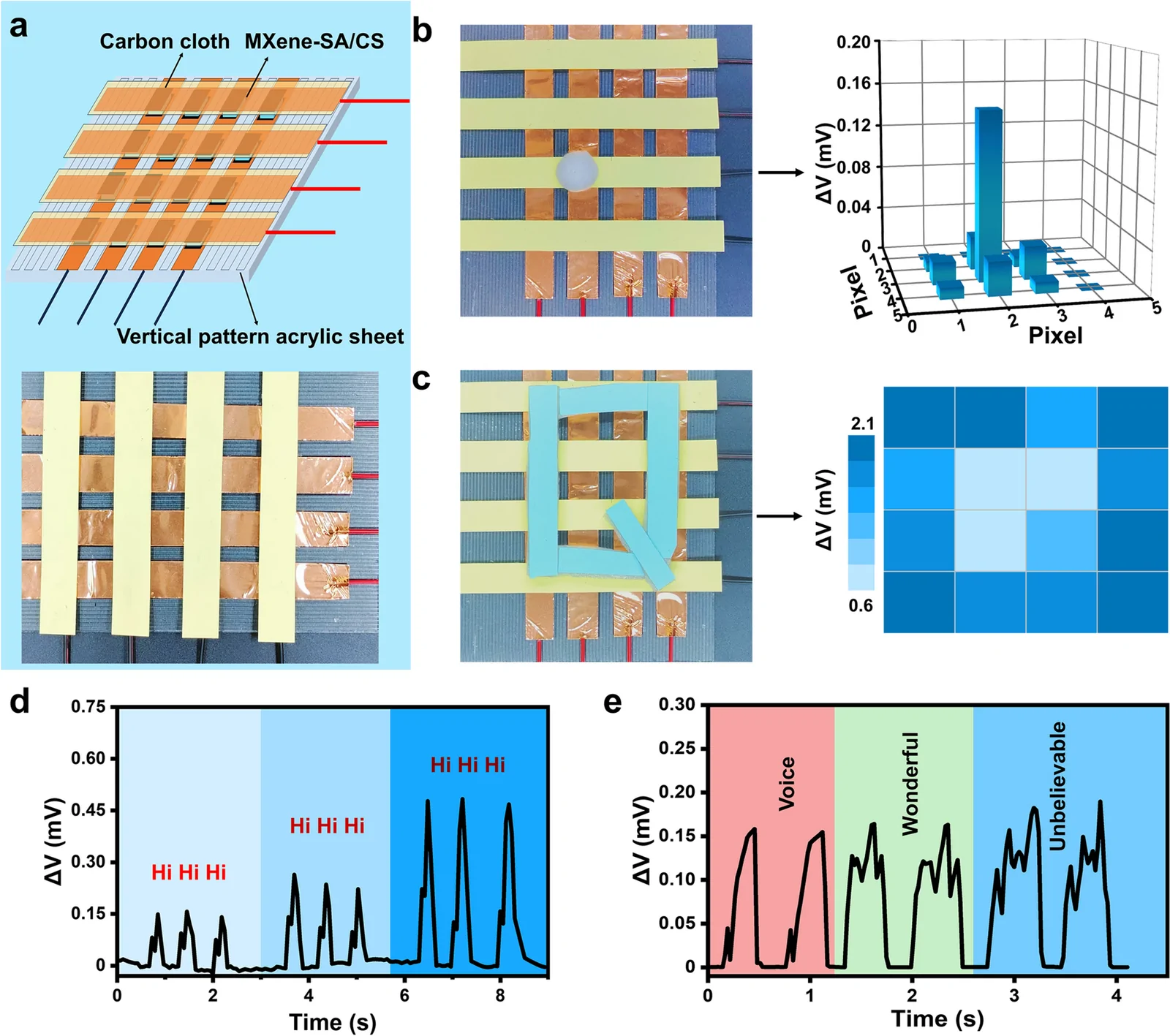
**a** Schematic diagram and actual optical photograph of a 4 × 4 pixel ion array assembled using vertically grooved orthogonal carbon cloth electrodes. **b** Potential changes were monitored by placing cotton balls and** c** low-density polyethylene foam letter respectively on a 4 × 4 pixel ion array assembled with vertical groove orthogonal carbon cloth electrodes. **d** Potential varies with different volumes and** e** different pronunciations

We explore the application of MXene-SA/CS hydrogel in sound sensing and pressure imaging based on the interface-dependent self-powered I-skins. Figure 5a shows a 4 × 4 pixel ion array assembled using vertically grooved orthogonal carbon cloth electrodes. The changes in detection voltage across pixels were shown by placing cotton balls (≤ 0.05 kPa) (Fig. 5b) and the pressure on the low-density polyethylene foam letter I-skin array (≤ 0.1 kPa) (Fig. 5c), demonstrating the sensitive perception of the self-powered I-skin based on MXene-SA/CS hydrogel. Based on the interface-dependent properties of self-powered I-skins, we subsequently designed and fabricated a simple sound detection device to monitor the potential changes at different volume levels (Fig. 5d) and different pronunciations (Fig. 5e). When saying “Hi” at different volumes and saying “Voice,” “Wonderful,” and “Unbelievable” at the same volume, there are significant differences in the electrical signals of the self-powered I-skins based on interface dependencies. These results demonstrate the potential of MXene-SA/CS hydrogel-based self-powered I-skin for applications in real-time monitoring of physiological signals during human activities, sound signal recognition, and human–machine interaction.

## Conclusions

In summary, we report a facile diffusion-complexation strategy for fabricating self-oriented gradient MXene-SA/CS hydrogel films, which function effectively as self-powered I-skins with outstanding EMI shielding and pressure-sensing performances. This diffusion-complexation strategy can couple osmotic pressure gradient-induced in-plane self-orientation of MXene nanosheets and diffusion-complexation reaction-induced spontaneous formation of a charge gradient, realized by simply bringing a high-concentration solution of LMW CS polycation into contact with a mixture of MXene/SA mixture. Here, the LMW CS polycation serves a dual role: as a reactant that induces the formation of a gradient hydrogel matrix through diffusion and electrostatic complexation, and as an osmotic pressure regulator that promotes matrix compression and in-plane alignment of MXene nanosheets. The resulting charge gradient endows the material with self-powered sensing capability, while the in-plane self-oriented MXene nanosheets impart exceptional EMI shielding performance and high-pressure sensitivity. In consequence, the as-prepared I-skins exhibit a high EMI shielding effectiveness of 48 dB, along with outstanding self-powered sensing capacity that provides a high sensitivity (3.067 mV kPa^−1^), a broad sensing range (0.05–80 kPa), a rapid response (120–130 ms), and a low detection limit (50 Pa). This study establishes a versatile strategy for creating integrated systems that achieve a multifunctional synergy of high-performance sensing, self-powering, and exceptional EMI shielding within a unified platform.

## Supplementary Information

Below is the link to the electronic supplementary material.

Supplementary file1 (DOCX 29494 KB)
