# Supplementary material for: Self-Oriented Gradient Ionic Skins for Dual-Function Electromagnetic Shielding and Self-Powered Sensing
Source: Nanomicro Lett. 2026 Jun 26;18:415. doi: 10.1007/s40820-026-02255-z (PMC13309594; doi:10.1007/s40820-026-02255-z)
Supplement: Supplementary file 1 — Supplementary file1 (DOCX 29494 KB) [file 40820_2026_2255_MOESM1_ESM.docx]

Supporting Information for

**Self-Oriented Gradient Ionic Skins for Dual-Function Electromagnetic Shielding and Self-Powered Sensing**

Donghao Zhang^1#^, Xiaodan Wang^1#^, Na Pan^2,^ *, Wenxin Fan^1,^ * and Kunyan Sui^1,^ *

^1^College of Materials Science and Engineering, Key Laboratory of Marine Bio-based Fibers of Shandong Province, Key Laboratory of Shandong Provincial Universities for Advanced Fibers and Composites, Qingdao University, Qingdao 266071, P. R. China

^2^College of Materials Science and Engineering, Liaocheng University, Liaocheng 252000, P. R. China

#Donghao Zhang and Xiaodan Wang contributed equally to this work.

*Corresponding authors. E-mails: [napan@lcu.edu.cn](mailto:napan@lcu.edu.cn) (Na Pan); [fanwenxin@qdu.edu.cn](mailto:fanwenxin@qdu.edu.cn) (Wenxin Fan); [sky@qdu.edu.cn](mailto:sky@qdu.edu.cn) (Kunyan Sui)

# **S1 Supplementary Notes**

**Note S1 Theoretical calculation of orientation parameters**

Based on the Hermans orientation function, the orientation degree of MXene in the MXene-SA/CS hydrogel was calculated [S1].

$\text{ρ}\text{ }\text{=}{I_{max}}/{I_{min}}$ (S1)

$\bar{{cos}^{2} \theta}\text{=}{\text{(1}-\text{ρ}\text{)}}/{\text{(1+2}\text{ρ}\text{)}}$ (S2)

$f=\frac{3\bar{{cos}^{2} \theta}-1}{2}$ (S3)

where *ρ* is the depolarization ratio, *I_max_* and *I_min_* are the maximum and minimum values of the intensity components at different angles in the scattered light (or fluorescence), *f* is the Hermann orientation function value, *θ* is the angle between the axis of a single molecular chain and the reference direction, and $\bar{{cos}^{2} \theta}$ represents the average value of *cos*² *θ* for all molecular chains, respectively.

**Note S2 Simulation process for self-polarized potential of gradient hydrogel**

Based on the Poisson-Nernst-Planck (PNP), the self-polarized potential of self-oriented gradient MXene-SA/CS hydrogel was numerically simulated [S2].

$\Delta\varphi=-\frac{\rho}{\varepsilon_{0}\varepsilon_{r}}=-\frac{e_{0}z_{i}c_{i}}{\varepsilon_{0}\varepsilon_{r}} (ⅈ=\mathrm{Na}^{+},\mathrm{CH}_{3}\mathrm{COO}^{-})$ (S4)

$\frac{\partial C_{i}}{\partial t}=-\nabla\cdot J_{i}=D_{i}\nabla^{2}c_{i}+\mu\nabla\cdot\left( c_{i}\nabla\varphi\right) (i=\mathrm{Na}^{+})$ (S5)

where *φ* is the local electric potential, *ε*_0_ and *ε_r_* are the vacuum permittivity and relative dielectric constant, *t* is time step, *z_i_*, *c_i_*, *J_i_*, *D_i_* and *μ_i_* are the charge number, local concentration, flux, diffusivity, and mobility of species *i*, respectively.

The space charges include fixed anions (-CH_3_COO^-^) on SA and mobile counter cations (Na^+^). We assume that, in the initial stage, the concentrations of Na^+^ and -CH_3_COO^-^ along the thickness directions are linearly distributed, ranging from 1 to 100 M. The concentration distribution of -CH_3_COO^-^ is fixed, but the Na^+^ diffuses spontaneously from the high-density side to the low-density side. A built-in potential can thus be formed. The initial condition in the simulation is defined as:

$c=1.98x+1 (0\leq x\leq l)$ (S6)

where *c* is the concentrations of Na^+^, and *l* is the thickness of the gradient hydrogels. The *l* is set as 50 μm in the simulation.

After the self-induced potential of gradient hydrogels is stabilized, the boundary conditions of Equations (S4 and S5) are defined as.

$c\left( x=0 \right)=c_{1}, c\left( x=l \right)=c_{2}$ (S7)

$\phi\left( x=0 \right)=0, \phi\left( x=l \right)=V_{0}$ (S8)

where *c_1_* and *c_2_* are the concentrations of Na^+^ at two surfaces of gradient hydrogel after its self-induced potential is stabilized, and *V*_0_ is the self-induced built-in potential voltage of gradient hydrogels. In the simulation, *c_1_*, *c_2_* and *V_0_* are set as 2 M, 98 M, and 32 mV, respectively.

**Note S3 Formation mechanism of built-in potential of gradient** **hydrogels**

The migration of ions within gradient polyelectrolyte hydrogels is driven by two forces, namely the concentration gradient-induced ionic diffusion and internal electric field-induced ionic drifting. The movement of a certain species *i* inside the hydrogel can be described by the Nernst-Planck equation:

$j_{i}=j_{dif-i}+j_{dri-i}=-D_{i}\left( \nabla c_{i}+\frac{z_{i}Fc_{i}}{RT}\nabla\phi\right)$ (S9)

where *j_i_*, *j_dif-i_*, and *j_dri-i_* are the total ionic flux density, diffusion-induced ionic flux density, and internal electric field-induced ionic flux density of *i*, respectively.

The stable *φ* can be obtained when the diffusion and drifting effects reach a dynamic balance (i.e., *j_i_* = 0 or *j_dif-i_* = -*j_dri-i_*). Thus, the built-in potential of gradient polyelectrolyte hydrogel can be represented as:

$\Delta\phi=\frac{\text{RT}}{z_{i}F}ln\frac{c_{i}\left( h \right)}{c_{i}\left( l \right)}$ (S10)

where *c_i_*(*h*) and *c_i_*(*l*) are the concentrations of *i* at the high charge density (HD) side and at low charge density (LD) side of gradient hydrogel, respectively.

**Note S4 The relationship between charge gradient and sensing performance**

The gradient modulation experiments are crucial for establishing a clearer structure—property relationship and for better elucidating the sensing mechanism. In this manuscript, we prepared a gradient MXene-SA/CS hydrogel using the diffusion-complexation method, but the charge gradient in this system is difficult to precisely control or modulate. Therefore, we fabricated two other types of hydrogels with tunable charge gradients to simulate the sensing performance of the MXene-SA/CS hydrogel-based I-skins and to quantitatively establish the relationship between the charge gradient and sensing performance (Fig. S17):

(1) Three-layer sodium alginate/polyacrylamide (SA/PAM) gradient hydrogels: These hydrogels consist of three SA/PAM hydrogel layers with different SA contents, allowing precise modulation of the charge gradient by adjusting the SA concentration in each layer. Three SA concentration gradients from the bottom layer to the top layer were employed, including: 0 wt%-1.0 wt%-2.0 wt%, 0.25 wt%-1.0 wt%-1.75 wt%, and 0.5 wt%-1.0 wt%-1.5 wt%. Clearly, a larger charge gradient (i.e., larger SA concentration gradient) leads to the higher output voltage and self-polarized potential of gradient hydrogels (Fig. S17a, and Eq. 8), as well as the greater pressure sensing sensitivity of resulting self-powered I-skins (Fig. S17a, b).

(2) Gradient P(AM-co-AMPS) polyanion hydrogels: These were prepared by electric field-assisted radical polymerization in a laboratory-made mold consisting of two indium tin oxide (ITO) conductive glasses separated by a spacer (see Fig. S17 c). The precursor solution contained acrylamide (AM, monomer), 2-acrylamido-2-methylpropanesulfonic acid (AMPS, anionic monomer), N,N'-methylenebisacrylamide (MBAA), ammonium persulfate (APS), and tetramethylethylenediamine (TEMED). Under a DC electric field, the negatively charged AMPS migrated directionally toward the anode, while the copolymerization of AM and AMPS formed a chemically crosslinked P(AM-co-AMPS) network that anchored the gradient of AMPS (or -SO_3_^-^ groups) within the hydrogel. The gradient of -SO_3_^-^ groups can be readily modulated by adjusting the applied direct current (DC) voltage. Specifically, increasing the DC voltage enhances the gradient of -SO_3_^-^ groups, as reflected by the increased sulfur content on the anode side and the decreased sulfur content on the cathode side (Fig. S17d, e). Similarly, enlarging the charge gradient (i.e., the gradient of -SO_3_^-^ groups) increases the output voltage, the self-polarized potential of the gradient hydrogels, and the pressure sensing sensitivity of the resulting self-powered I-skins (Fig. S17f, g).

Collectively, these two gradient modulation experiments demonstrate that increasing the gradient of charged groups enhances the self-polarized potential of the hydrogel, which in turn benefits higher pressure sensing sensitivity (Fig. S17).

**Note S5** **Fabrication of the gradient SA/PAM hydrogel and P(AM-co-AMPS) hydrogel**

The SA/PAM hydrogels were prepared using a thermally induced free radical polymerization method. In brief, AM (monomer, 20 g), SA (0.25-2.0 g), and MBAA (crosslinking agent, 34.5 mg) were added into 80 mL of distilled water. After stirring for 0.5 hours, APS (initiator, 32 mg) and TEMED (accelerator, 80 μL) were added to the solution under ice bath conditions. The mixture was sonicated in ice water for 2 minutes, and then injected into sealed molds with different configurations. The SA/PAM hydrogel layers with a thickness of 0.4 mm were obtained after thermal polymerization in an oven at 50 °C for 3 hours. The three-layer SA/PAM gradient hydrogels were constructed just by stacking the obtained SA/PAM hydrogel layer.

The P(AM-co-AMPS) hydrogels were prepared by electric field-assisted free radical polymerization. The brief procedure is as follows: AM and AMPS (30 g in total) and MBAA (0.02 g) were first dissolved in 70 g of distilled water. After stirring for 0.5 h in an ice-water bath, APS (0.05 g) and 100 μL of TEMED were sequentially added to the solution. The resulting mixed solution was ultrasonically treated in an ice-water bath for 5 min and then injected into a sealed mold composed of ITO conductive glass plates. Subsequently, different direct current (DC) voltages (0.5-1.5 V) were applied to the ITO glass plates of the mold. The samples were left to stand at room temperature for 30 min and then placed in an oven at 50 °C for 3 h. The DC voltage was maintained only during the first hour and then turned off for the remaining reaction time (Fig. S17c).

# **S2 Supplementary Figures**


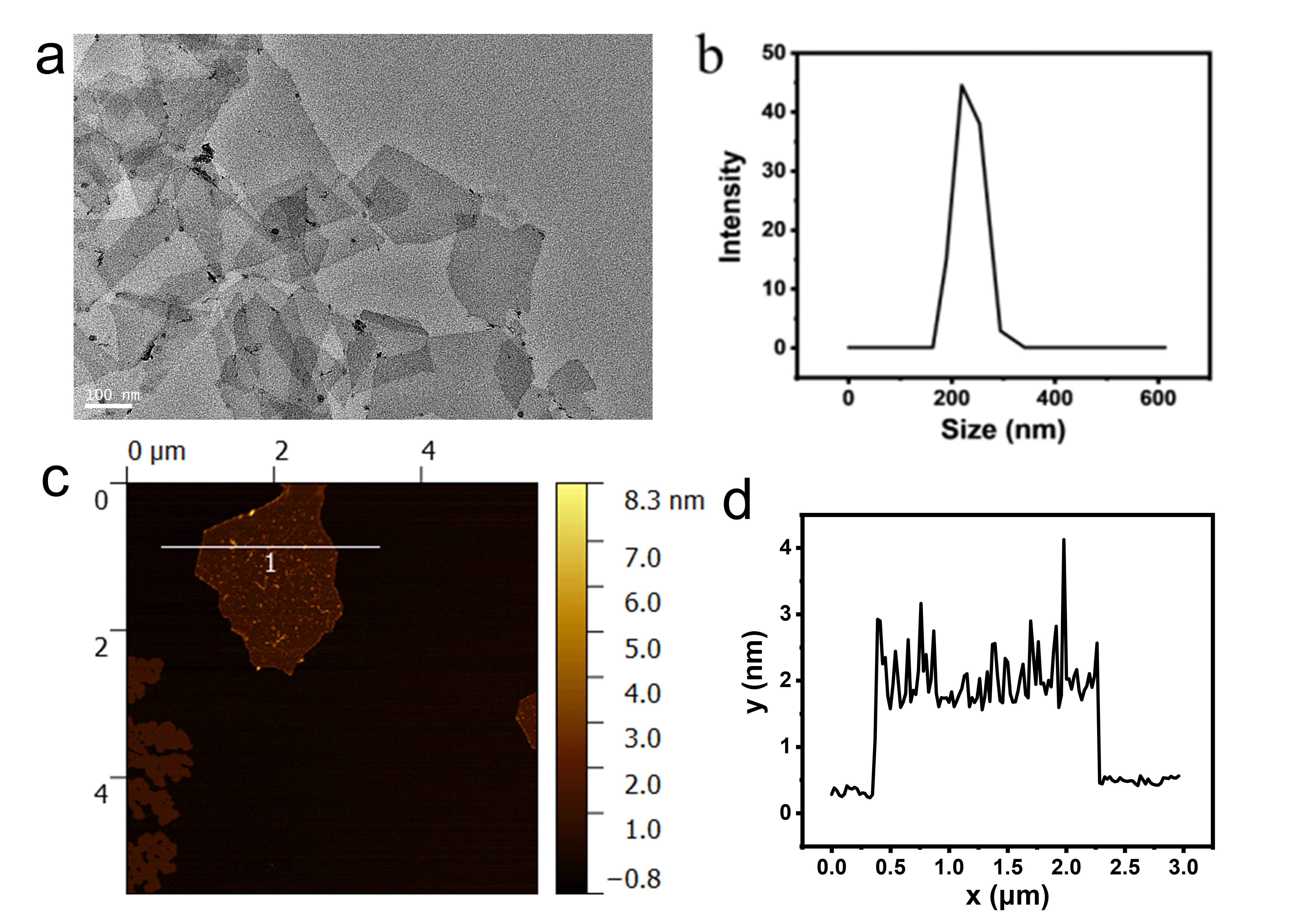


**Fig. S1** **a** TEM image, **b** dynamic light scattering curve, **c** AFM image, and **d** thickness distribution curve of as-prepared Ti_3_C_2_T_x_ MXene.


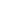


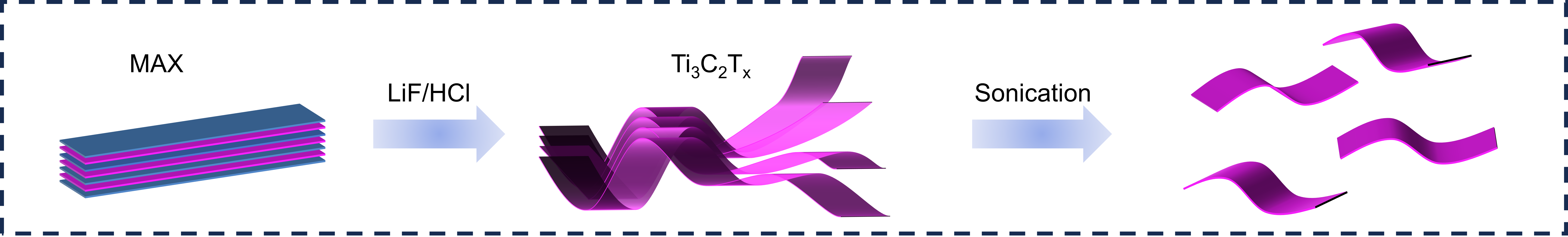


**Fig. S2** Schematic illustration of the preparation process of Ti_3_C_2_T_x_ MXene from Ti_3_AlC_2_.


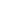

**Fig. S3** Osmotic pressure of LMW CS solution and MXene/SA mixture solution.

**Fig. S4** Variation of the thickness of MXene-SA/CS hydrogel film over reaction time.


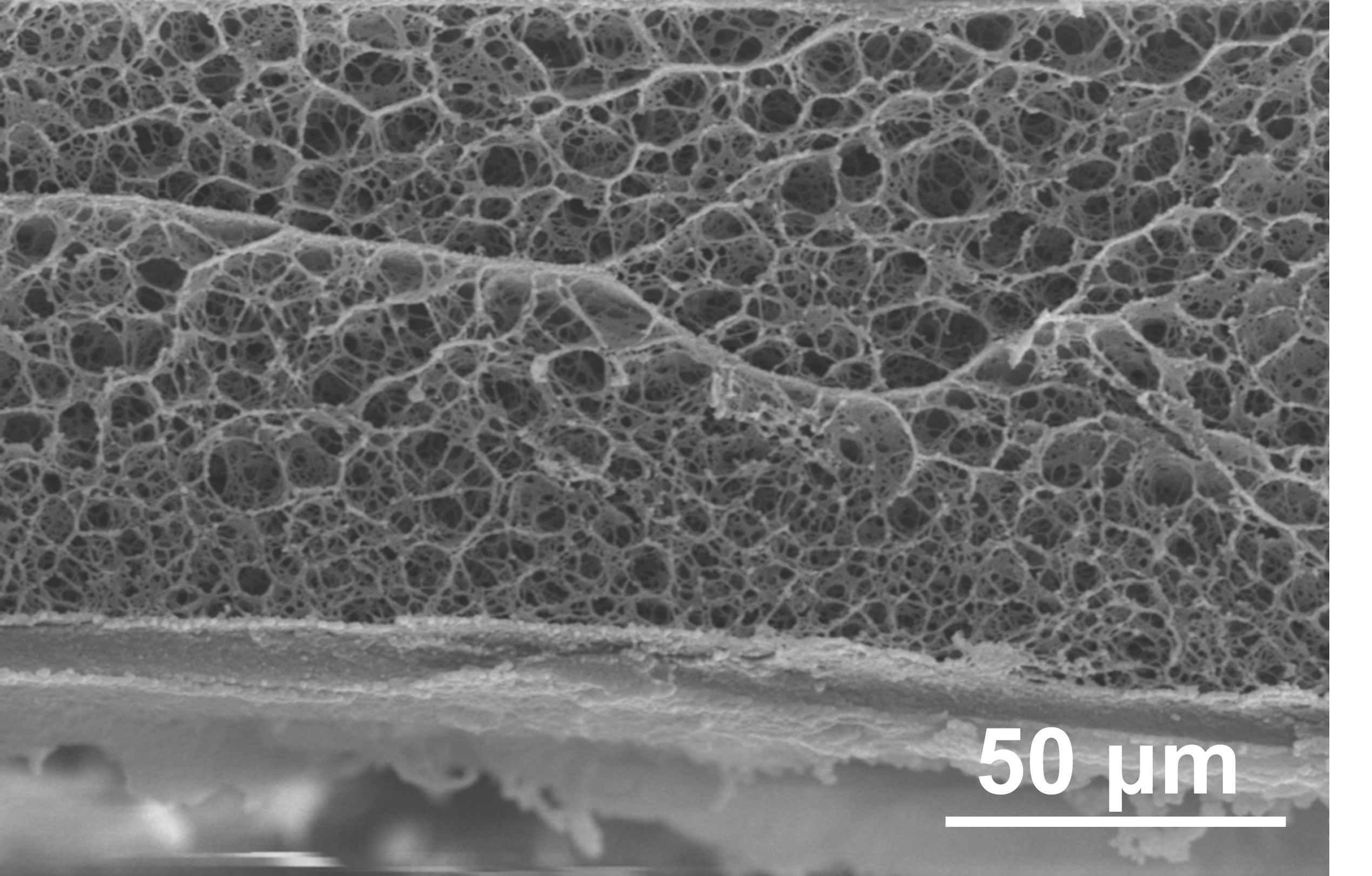


**Fig. S5** Cross-sectional SEM images of SA/CS hydrogel.

**Fig. S6** The polarized Raman spectra of the MXene-SA/CS hydrogel at different angles.

*
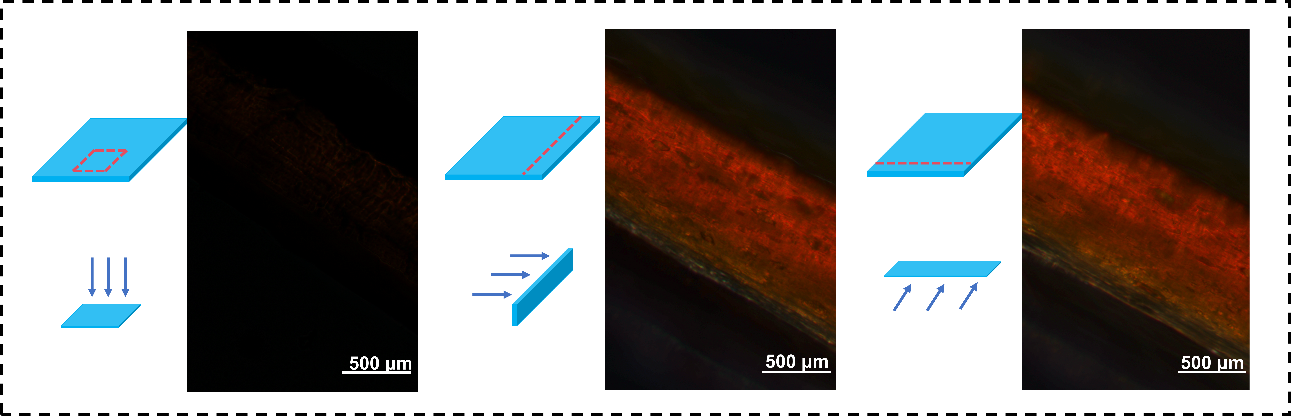
*

**Fig. S7** POM images of SA/CS hydrogel observed from different directions.

**Fig. S8** Raman spectra recorded at various positions of the MXene-SA/CS hydrogel.

**Fig. S9** XPS spectra of the HD side and LD side of the self-oriented gradient MXene-SA/CS hydrogel.

**Fig. S10** Content of CS and SA at the HD side and LD side.

**Fig. S11** Viscosity of MXene/SA mixtures at different concentrations.

**Fig. S12** Conductivity of MXene-SA/CS hydrogels prepared with different MXene contents.
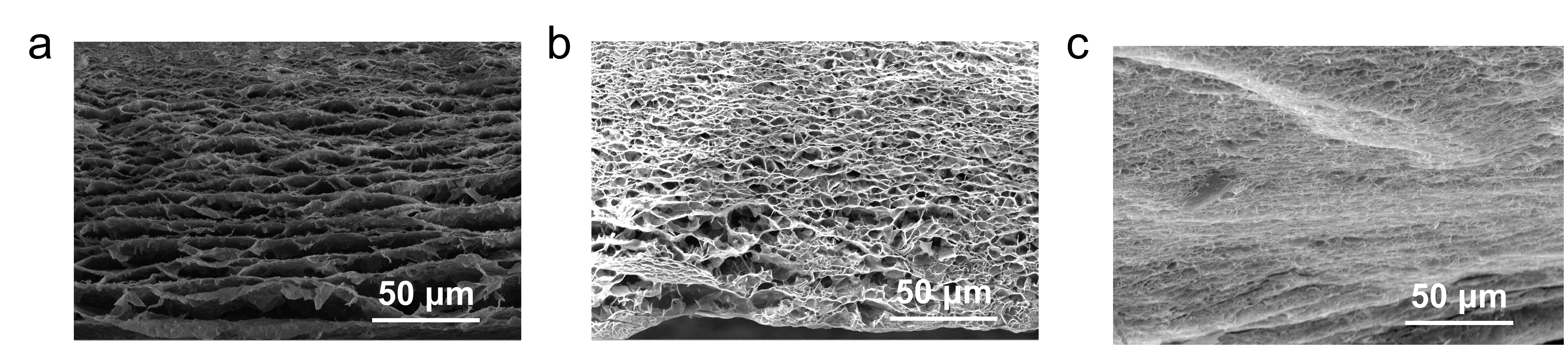


**Fig. S13** Cross-sectional SEM images of MXene-SA/CS hydrogel prepared with different SA contents: **a** 0.25 wt%, **b** 0.5 wt% and **c** 0.75 wt%.


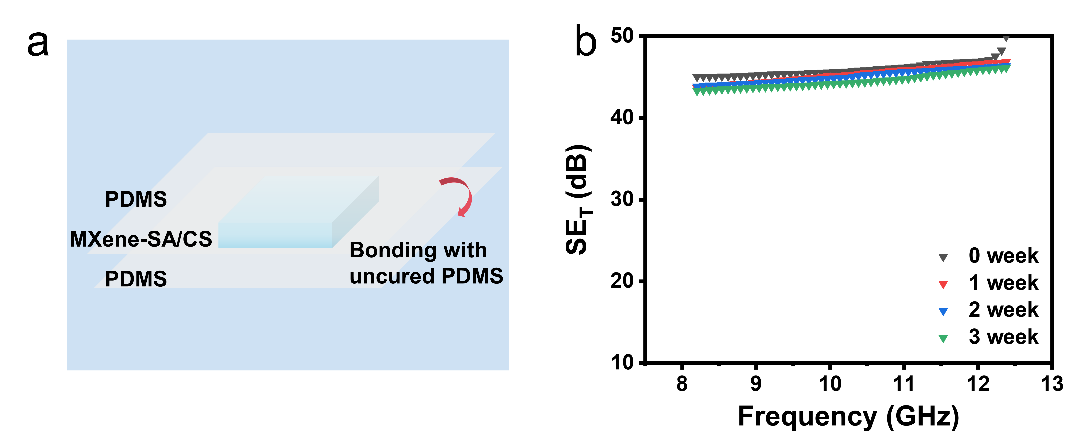


**Fig. S14 a** Schematic illustration of the encapsulation of MXene-SA/CS hydrogel with commercial PDMS membranes. **b** SE_T_ of MXene-SA/CS hydrogels after storage in ambient air for different times.

**Fig. S15** EMI shielding performance with incident electromagnetic waves respectively exposed to the HD side and the LD side.


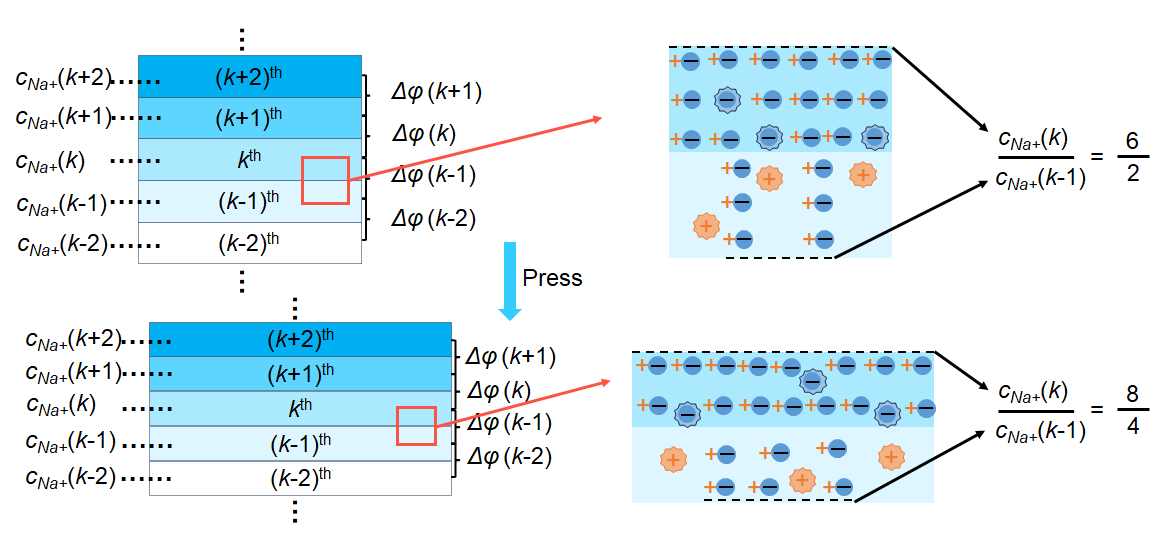


**Fig. S16** Schematic illustration of thickness-dependent self-polarized potential of gradient polyelectrolyte hydrogels [S3].


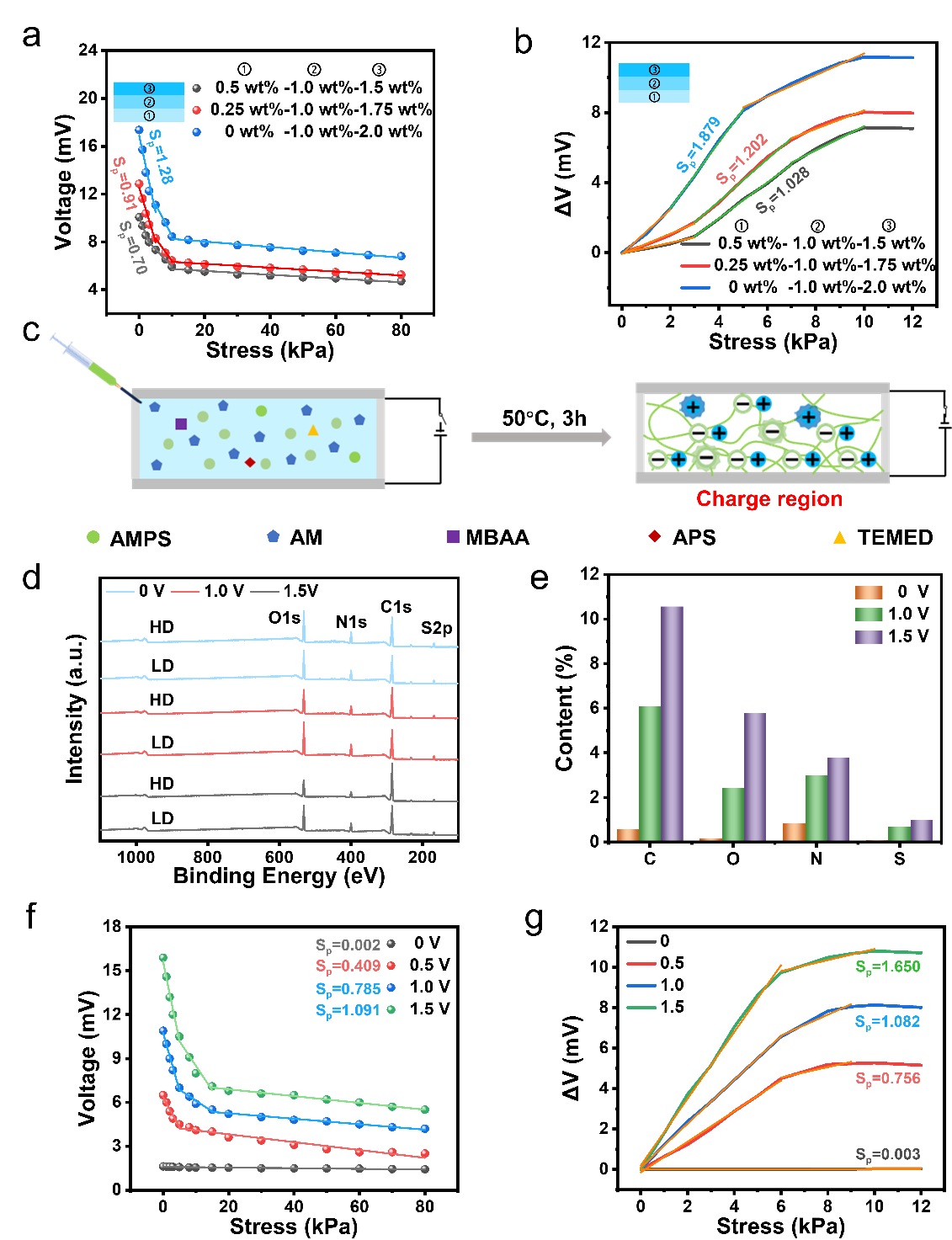


**Fig. S17** The relationship between charge gradient and sensing performance. Sensing sensitivity of thickness-dependent **a** and interface-dependent **b** three-layer SA/PAM hydrogels with different SA gradients. **c** Schematic illustration of the fabrication of gradient P(AM-co-AMPS) hydrogel. **d** XPS spectra and **e** atomic percentage of C, O, N and S on the HD and LD sides of gradient P(AM-co-AMPS) hydrogels prepared under different DC voltages. Sensing sensitivity of **f** thickness-dependent and **g** interface-dependent P(AM-co-AMPS) hydrogels prepared under different DC voltages.


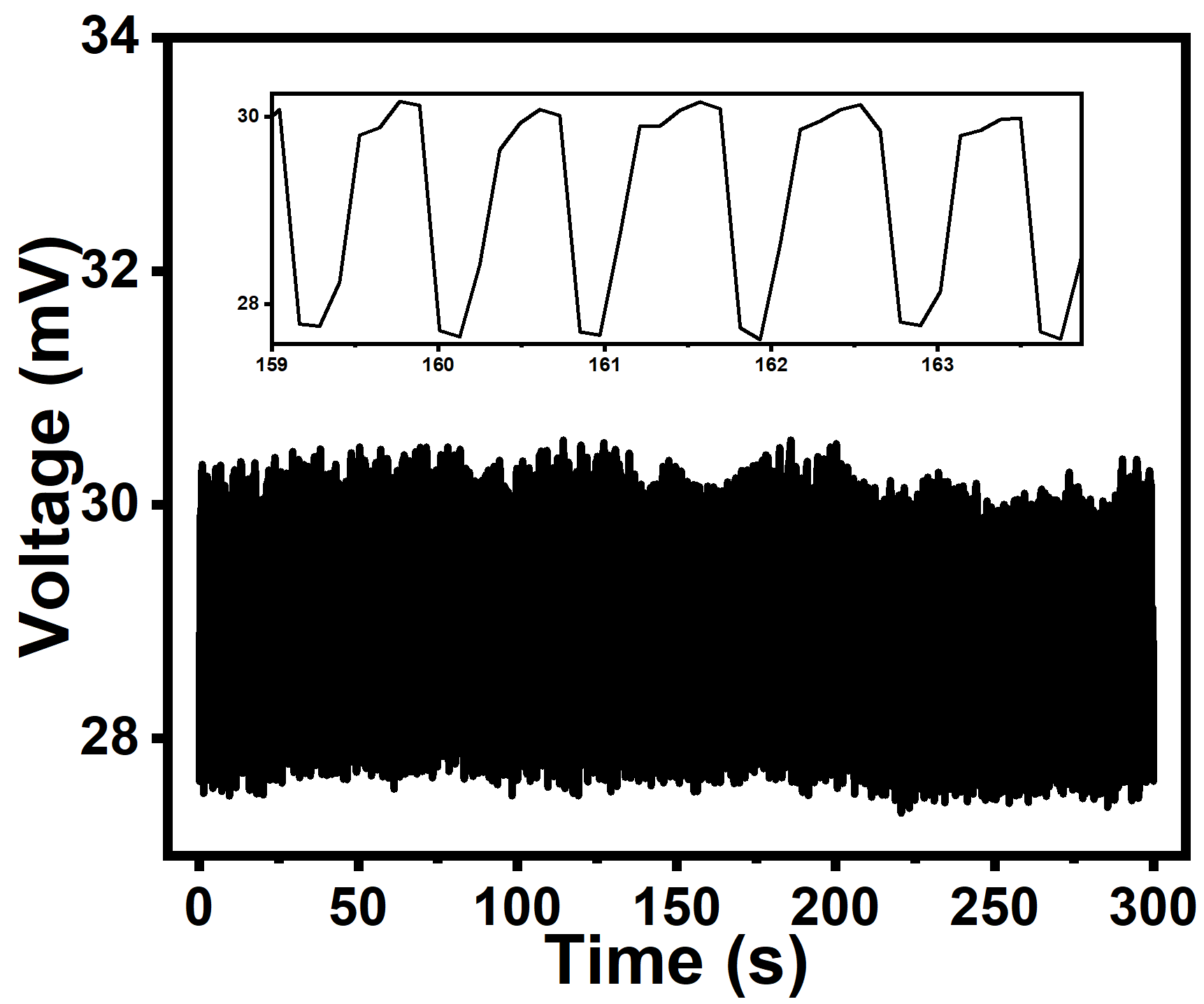


**Fig. S18** Open-circuit voltage of thickness-dependent self-powered I-skin in response to the repeated loading and unloading of 20 kPa pressure for 200 cycles.

**Fig. S19** Comparison of the sensitivity between previously reported gradient hydrogel-based self-powered I-skins and our self-powered I-skin [S3-S7].


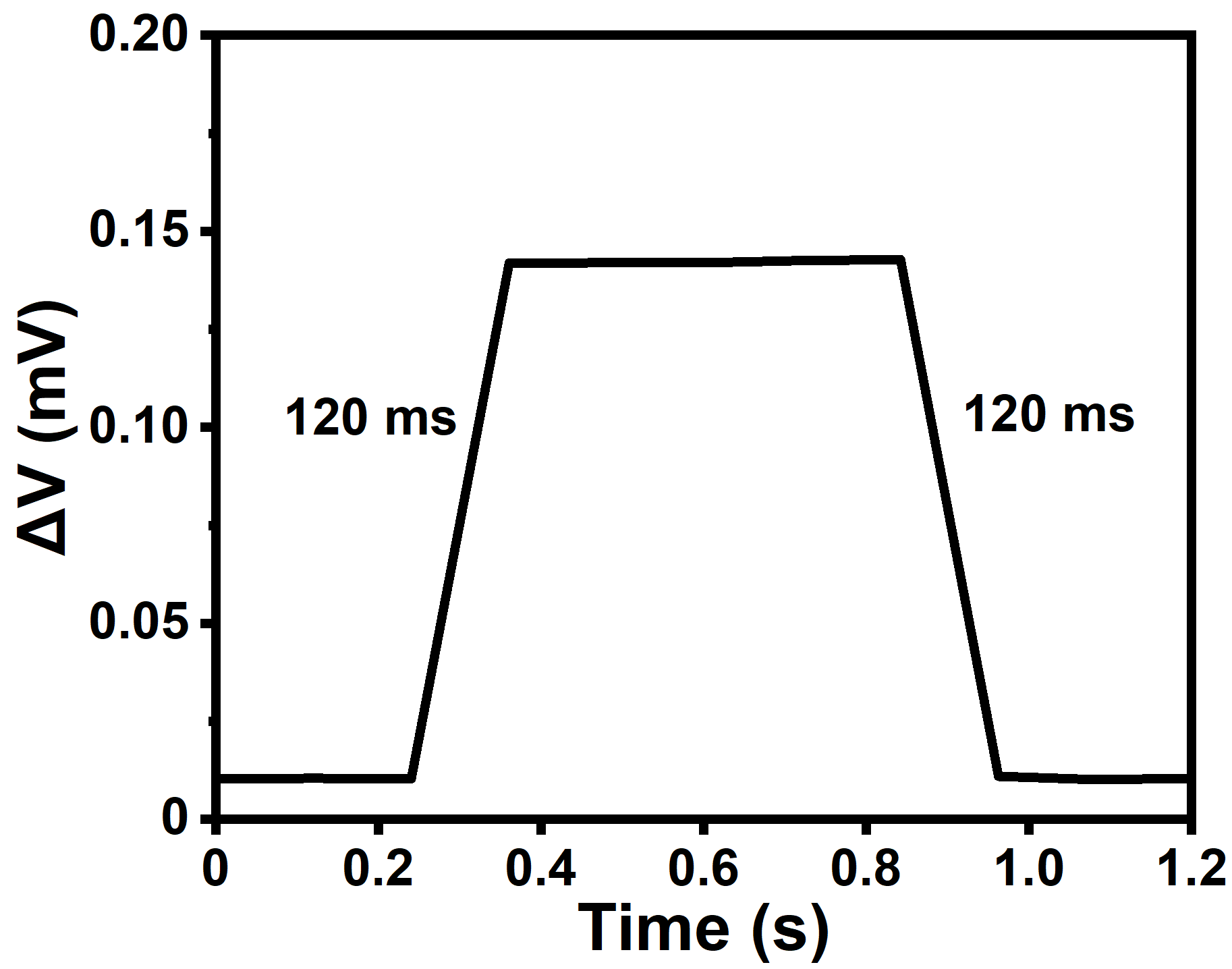


**Fig. S20** Response/recovery time of the interface-dependent self-powered I-skin in response to 0.05 kPa pressure.


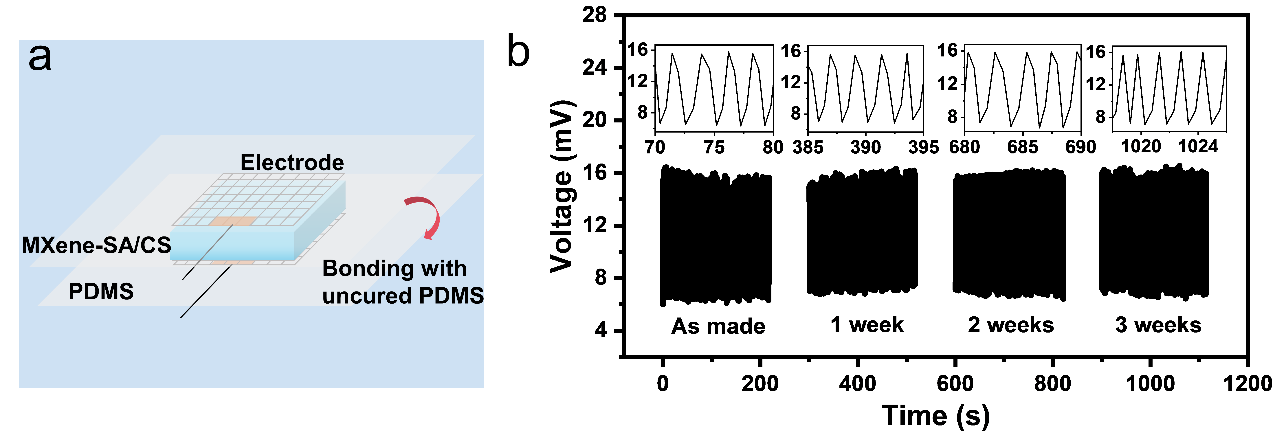


**Fig. S21** **a** Schematic illustration of the encapsulation of gradient MXene-SA/CS hydrogel-based self-powered I-skin. **b** Long-term cyclic stability of the self-powered I-skin in response to repeated loading/unloading at 2 kPa after storage in ambient air for different times.


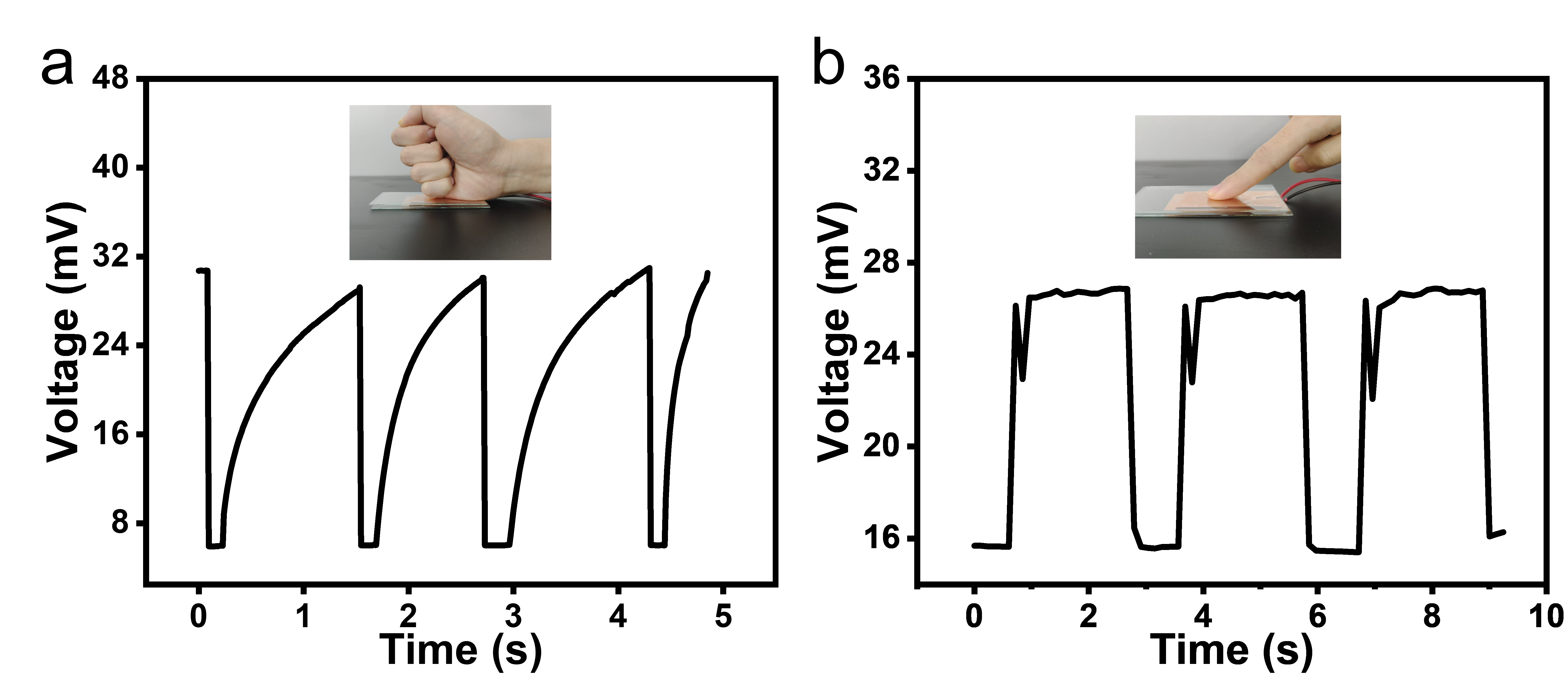


**Fig. S22** Voltage changes of self-powered I-skins in response to **a** the intense pressing and **b** the gentle pressing.

# **S3 Supplementary Table**

**Table S1** Systematic comparison of the key properties of MXene-based hydrogels and polyelectrolyte gradient hydrogels

| Samples | Filler (wt%) | EMI | SE_T_ (dB) | Self-powering | Sensitivity (mV/kPa) | Strength/Stretchability (MPa/%) |
| --- | --- | --- | --- | --- | --- | --- |
| PPM [S8] | 5 | √ | 59.3 | **×** | — | 0.126/100 |
| Ca-PAA-CNF-MXene [S9] | 10 | √ | 30 | **×** | 2.16 | —/1000 |
| CCM [S10] | 1.2 | √ | 51.8 | **×** | — | 0.5/424 |
| PMPχ-SSD-PCOH [S11] | 1.8 | √ | 38.7 | **×** | — | 0.2/60 |
| M1-P4 [S12] | 1.0 | √ | 51.7 | **×** | — | 0.12/5 |
| MXene-PAA-ACC [S13] | 8.5 | √ | 45.3 | **×** | — | 0.02/1500 |
| PAM/MSA [S4] | — | **×** | — | √ | 0.116 | 0.07/1150 |
| GP [S5] | — | **×** | — | √ | 0.175 | 0.125/8 |
| GPMs [S3] | — | **×** | — | √ | 0.329 | 3.0/40 |
| PMIA/SPSF-PMIA [S6] | — | **×** | — | √ | 0.512 | 25.8/23 |
| PAM/MSA-PAM/MCS [S7] | — | **×** | — | √ | 0.679 | 0.06/900 |
| This work | 1.0 | √ | 48 | √ | 3.067 | 0.24/7 |

**Supplementary References**

1. E. Brandley, E.S. Greenhalgh, M.S.P. Shaffer, Q. Li, Mapping carbon nanotube orientation by fast Fourier transform of scanning electron micrographs. Carbon **137**, 78–87 (2018). <https://doi.org/10.1016/j.carbon.2018.04.063>
2. H. Wang, M. Du, H. Jiang, R. Zhou, Y. Wang et al., Gradient p-polyanion/n-polycation heterojunction endows ionic diodes with vastly boosted output voltage, power density and sensitivity. Adv. Funct. Mater. **34**(45), 2407639 (2024). <https://doi.org/10.1002/adfm.202407639>
3. M. Xia, N. Pan, C. Zhang, C. Zhang, W. Fan et al., Self-powered multifunction ionic skins based on gradient polyelectrolyte hydrogels. ACS Nano **16**(3), 4714–4725 (2022). <https://doi.org/10.1021/acsnano.1c11505>
4. X. Zhu, P. Qi, W. Fan, H. Wang, K. Sui, Bioinspired multifunctional self-powered ionic receptors derived by gradient polyelectrolyte hydrogels. Chem. Eng. J. **438**, 135610 (2022). <https://doi.org/10.1016/j.cej.2022.135610>
5. S. Shen, C. Liu, W. Fan, K. Sui, A self-detection mechanism toward stable multiple perception of ionic skins. Adv. Funct. Mater. **35**(25), 2423915 (2025). <https://doi.org/10.1002/adfm.202423915>
6. B. Liang, W. Fan, K. Sui, Ultrastrong and heat-resistant self-powered multifunction ionic sensor based on asymmetric meta-aramid ionogels. Chem. Eng. J. **519**, 165332 (2025). <https://doi.org/10.1016/j.cej.2025.165332>
7. M. Du, D. Zhang, W. Fan, K. Zhao, Y. Xia et al., Ionic diode-based self-powered ionic skins with multiple sensory capabilities. Mater. Today Phys. **26**, 100744 (2022). <https://doi.org/10.1016/j.mtphys.2022.100744>
8. Q. Yan, Z. Liu, J. Xiong, H. Lian, H. Chen et al., Stimuli-responsive MXene/PNIPAM hydrogel WITH high-performance and tunable electromagnetic interference shielding performance. Adv. Sci. **12**(31), e05551 (2025). <https://doi.org/10.1002/advs.202505551>
9. J. Wei, C. Zhu, Z. Zeng, F. Pan, F. Wan et al., Bioinspired cellulose-integrated MXene-based hydrogels for multifunctional sensing and electromagnetic interference shielding. Interdiscip. Mater. **1**(4), 495–506 (2022). <https://doi.org/10.1002/idm2.12026>
10. H. Sima, B. Liu, X. Shi, C. Zhang, CNT@CNF/MXene hydrogel with complete conductive network for flexible anti-freeze sensor and electromagnetic shielding. Carbohydr. Polym. **366**, 123825 (2025). <https://doi.org/10.1016/j.carbpol.2025.123825>
11. T.-Y. Zhu, W.-J. Jiang, S. Wu, Z.-J. Huang, Y.-L. Liu et al., Multifunctional MXene/PEDOT: PSS-based phase change organohydrogels for electromagnetic interference shielding and medium-low temperature infrared stealth. ACS Appl. Mater. Interfaces **16**(12), 15372–15382 (2024). <https://doi.org/10.1021/acsami.4c01001>
12. J. Liu, L. McKeon, J. Garcia, S. Pinilla, S. Barwich et al., Additive manufacturing of Ti_3_C_2_-MXene-functionalized conductive polymer hydrogels for electromagnetic-interference shielding. Adv. Mater. **34**(5), 2106253 (2022). <https://doi.org/10.1002/adma.202106253>
13. Y. Zhu, J. Liu, T. Guo, J.J. Wang, X. Tang et al., Multifunctional Ti_3_C_2_T*_x_* MXene composite hydrogels with strain sensitivity toward absorption-dominated electromagnetic-interference shielding. ACS Nano **15**(1), 1465–1474 (2021). <https://doi.org/10.1021/acsnano.0c08830>
